# Brushite-modified titanium surface elicits early osteointegrative gene expression signatures in human mesenchymal stem cells: insights from NGS analysis

**DOI:** 10.3389/fbioe.2026.1943293

**Published:** 2026-09-23

**Authors:** Lavinia Raimondi, Viviana Costa, Gianluca Zappini, Marzio Piccinini, Alessia Gallo, Nicola Cuscino, Claudia Carcione, Lia Pulsatelli, Luca Cattini, Angela De Luca, Daniele Bellavia, Angelo Toscano, Gianluca Giavaresi

**Affiliations:** 1 Scienze e Tecnologie Chirurgiche, IRCCS Istituto Ortopedico Rizzoli, Bologna, Italy; 2 Lincotek Medical, Trento, Italy; 3 Dipartimento di Ricerca, IRCCS ISMETT (Istituto Mediterraneo per i Trapianti e Terapie ad Alta Specializzazione), Palermo, Italy; 4 Fondazione Ri.MED, Palermo, Italy; 5 Laboratory of Immunorheumatology and Tissue Regeneration, IRCCS Istituto Ortopedico Rizzoli, Bologna, Italy; 6 Ortopedia Generale, IRCCS Istituto Ortopedico Rizzoli, Bologna, Italy

**Keywords:** bioactive biomaterials, brushite, early transcriptional response, mesenchymal stem cells, RNA-seq, titanium implants

## Abstract

Brushite (dicalcium phosphate dihydrate) is a resorbable coating with significant potential to enhance implant osseointegration. However, the early molecular mechanisms through which it directs mesenchymal stem cell (MSC) behavior remain poorly understood. This study investigates the early transcriptional response of human MSCs cultured on brushite-coated Ti6Al4V compared to uncoated titanium at 7 days, a critical regulatory window that precedes overt differentiation. By employing both basal and osteogenic culture conditions, we decoupled material-specific effects from soluble biochemical cues. ICP-OES confirmed a high-purity Ca/P-based coating. RNA-seq analysis identified 390 and 613 differentially expressed genes under osteogenic and basal conditions, respectively, demonstrating a robust material-driven response. Pathway enrichment revealed the selective involvement of pro-osteogenic signaling (BMP, Wnt, Hedgehog), metabolic/mitochondrial reprogramming, and a controlled inflammatory response. Conversely, the downregulation of proliferation- and mechanotransduction-related genes is consistent with a transition toward a stabilized, differentiation-permissive state. Notably, a 25-gene core signature was identified across all conditions, representing a stable material-dependent transcriptional fingerprint. These findings were validated by qRT-PCR and corroborated at the protein level via multiplex assays (Bio-Plex), showing increased secretion of extracellular matrix and pro-regenerative factors. Collectively, our findings suggest that brushite is not merely a passive scaffold but promotes an early transcriptional program in MSCs consistent with osteogenic commitment, highlighting its potential to accelerate the clinical performance of bioactive implants.

## 1 Introduction

Titanium implants, widely used in orthopedic and dental applications, offer excellent biocompatibility and mechanical properties. However, their ability to achieve osseointegration, i.e., the bond between the implant and bone tissue, is strongly influenced by the surface characteristics. Surface modification tailors the implant interface to optimize biological interactions and promote efficient osseointegration (Bagno et al., 2004; Abdo et al., 2025).

Key surface modification techniques include: 1) Surface roughening (mechanical methods like sandblasting or chemical methods like acid etching), which increases surface area for cells attachment, improving bone-implant contact; 2) Metallic coatings (e.g. welded beads, mesh, plasma spray coatings), mainly made of titanium, which create a rougher surface and eventually a layer with interconnected pores, ideal for long-term fixation; 3) Coating with bioactive materials (e.g., hydroxyapatite, brushite, or calcium phosphates), which mimic bone mineral components and enhance biological activity; 4) Anodization and plasma electrolytic oxidation, which create a thicker, nano-structured oxide layer to promote cells adhesion and can be functionalized for further bioactivity; 5) Hydrophilic treatments, which make the surface water-attractive, improving protein adsorption and cells attachment (Wu et al., 2025; Le Guéhennec et al., 2007; Xue et al., 2020). These modifications are critical for promoting osseointegration, the direct bond between living bone and the implant, which is essential for the long-term success of orthopedic, dental, and spinal implants Successful osseointegration requires biocompatible, non-toxic surfaces that actively support bone cell adhesion, differentiation, and integration. (Zhu et al., 2021; Novaes et al., 2010; Hao et al., 2021). Surface modification is therefore crucial to enhance bone healing and implant stability while reducing fibrous tissue formation, implant loosening, inflammation, infection, and overall implant failure (Zhu et al., 2021; Zhang et al., 2025; Hotchkiss et al., 2016; Boyan et al., 2016). Ongoing research aims to clarify how surface roughness, coatings, chemical composition, and wettability influence the behavior of mesenchymal cells and osteoblasts, including cell adhesion, proliferation, differentiation, and matrix formation (Boyan et al., 2016). Key molecular signaling pathways, such as growth factor–and integrin-mediated signaling, are being studied for their roles in osseointegration. However, the molecular basis of these responses remains largely unresolved, and surface modifications may modulate multiple biological pathways that influence the healing process. Additionally, while surface modifications often improve short-term osseointegration, their long-term effects remain uncertain. The lack of understanding of these mechanisms has led to empirical approaches in implant design, creating challenges in optimizing treatments and predicting outcomes. Therefore, while surface modification is critical to the success of titanium implants, the biological and molecular mechanisms underlying osseointegration remain complex and incompletely comprehended. Continued research is required to elucidate these mechanisms and to guide the optimization of implant design for safer, more effective, and more durable orthopedic devices. Among the surface modifications of greatest interest due to its biological characteristics and strong interaction with the surrounding tissue is electrochemically deposited dicalcium phosphate dihydrate, or brushite. This method is commonly used as a coating in orthopedic and dental implants due to its advantages over hydroxyapatite (HA) thermal spray coatings. These benefits include higher solubility, which releases more Ca^2^ and PO^3^ at the implant interface, lower-temperature production, and a non-line-of-sight application method that enables uniform coating of irregularly shaped implants (Ducheyne et al., 1993). Also, the application method based on electrochemical deposition is ideal for “complex” surfaces like those obtained by Additive Manufacturing, which can have intricate geometries and porosity where traditional (line-of-sight) coatings fail (Wu et al., 2025; Le Guéhennec et al., 2007; Xue et al., 2020). Brushite resorbs faster than HA, making it well suited for temporary surgical applications where controlled resorption supports healing and tissue integration. In bone repair, brushite-coated implants resorb more quickly, providing early mechanical support while enabling gradual replacement by native bone. As the material dissolves, new bone fills the space, allowing brushite to function as a temporary scaffold rather than a permanent implant (Lukina et al., 2023; Tamimi et al., 2008; Reigstad et al., 2007). This is particularly useful in cases such as bone fracture repair or bone defect regeneration, where the implant helps guide the healing process. Once healing progresses, the material resorbs, minimizing the long-term presence of foreign material. This process helps avoid issues like chronic inflammation, immune responses, or implant failure, which are more likely if the implant remains in the body permanently. The rapid resorption of brushite also lowers the risk of complications such as infection or loosening, as the material disappears once the healing process is complete. Additionally, as brushite resorbs, it promotes bone remodeling by creating a dynamic environment where new bone gradually replaces the material. In contrast, non-resorbable materials such as HA may sustain this process to a lesser extent, potentially influencing bone remodelling once healing is complete. Again, while the benefits of brushite coatings in promoting osseointegration are well established, the molecular and biological mechanisms driving these effects, such as specific cellular interactions, signaling pathways, and bone remodeling processes, are still not fully understood. This gap in knowledge presents a challenge, as deeper understanding is essential for further optimizing biocompatibility and performances of implants.

This study aims to address this gap in the current state of research and highlights the need for continued investigation to fully elucidate these processes. In our previous study (Raimondi et al., 2025), we showed that brushite-coated Ti6Al4V enhances bone healing and osseointegration by modulating the secretome of human mesenchymal stem cells (MSCs), selected for their multipotency, homing ability, and immunomodulatory functions. Brushite promoted the secretion of osteogenic factors, proteins involved in blood clotting, energy metabolism, mitochondrial biogenesis, stress responses, and chromatin-related proteins, suggesting a role for chromatin dynamics in bone regeneration (Raimondi et al., 2025). These findings identify brushite-coated Ti6Al4V as a promising scaffold for bone regeneration and warrant further investigation of its molecular mechanisms. While secretome analysis reveals protein-level changes, next-generation sequencing (NGS) provides a broader view of the transcriptional programs and pathways driving these responses. Integrating both approaches enables a comprehensive understanding of how brushite-coated titanium regulates regenerative signaling and functional protein expression. Here, we performed next-generation sequencing (NGS) of MSCs cultured for 7 days on brushite-coated or uncoated Ti6Al4V under both osteogenic and basal conditions to distinguish the effects of soluble differentiation cues from the intrinsic bioactivity of the brushite coating. The 7-day time point was selected to capture the early molecular events preceding overt osteogenic differentiation. Key osteogenic markers were validated by qRT-PCR, and Bio-Plex/Luminex assays assessed the corresponding protein changes. Overall, our findings reveal coordinated early transcriptional responses showing that brushite actively directs MSC commitment toward osseointegrative lineages rather than acting as a passive scaffold.

## 2 Materials and methods

### 2.1 Preparation of substrates

The specimen substrates (diameter 10 mm, thickness 3 mm) were fabricated by laser powder bed fusion (L-PBF) at Lincotek Medical facility (Italy), using a M290 machine (EOS GmbH, Germany) equipped with a 400 W laser source. These specimens were fabricated from a biomedical grade titanium alloy (Ti6Al4V) powder with particle sizes ranging from 15 to 45 μm (Ti64ELI, EOS GmbH, Germany). After fabrication, all specimens were subjected to a vacuum heat treatment to transform the initial α′-martensitic microstructure into a stable α+β phase, thereby relieving the residual stresses generated during the L-PBF process. Finally, the native AM surface was cleaned with a mixture of distilled water and commercial tetrasodium pyrophosphate (M 30, FIMM SAS, France).

### 2.2 Brushite electrodeposition process

The coatings were deposited by a pulsed cathodic galvanostatic technique at Lincotek Medical facility (Italy) using the Ti6Al4V disc as the cathode and a platinum-coated titanium mesh as the anode, immersed in a supersaturated calcium phosphate aqueous solution obtained by dissolving calcium phosphate monobasic monohydrate (Sigma Aldrich-Merck, Germany) in distilled water (30 g/L).

The electrolyte provides an electrical circuit between the electrodes in the cell. The deposition occurs under a pulsed electrical current (Raimondi et al., 2025).

During electrodeposition, the reactions described by the Equations 1-5 may occur at the surface of the cathode submerged in the electrolytic solution.

$$O_{2}+{2H}_{2}O+4e^{-}\to 4{\text{OH}}^{-}$$ (1)

$${2H}_{2}O+{2e}^{-}\to H_{2}+{2\text{OH}}^{-}$$ (2)

$${2H}^{+}+{2e}^{-}\to H_{2}$$ (3)

$$H_{2}{\text{PO}}_{4}^{-}+{\text{OH}}^{-}\to {\text{HPO}}_{4}^{2^{-}}+H_{2}$$ (4)

$${\text{HPO}}_{4}^{-}+{\text{OH}}^{-}\to {\text{PO}}_{4}^{3^{-}}+H_{2}$$ (5)

Equation 2 corresponds to the reduction of H_2_O which results in formation of hydrogen bubbles near the working electrode. As hydrogen is one of the products of Equations 2, 3, the local pH of the electrolyte solution in the vicinity of the cathode increases. The increased pH level provides a suitable substrate for acid–base reactions, as stated in Equation 4. An increase in concentration of OH- ions can lead to the increased number of hydrogen phosphate and phosphate ions through Equations 4, 5, i.e., reduction reactions of HPO_4_^2-^ and PO_4_^3-^.

Ions present in the electrolyte, such as Ca^2+^ and H^+^, move toward the cathode due to the electric field gradient, while existing HPO_4_^2-^ ions remain in the diffusing layer due to the differential concentration between the surface of cathode and the bulk electrolyte. Figure 1 schematically illustrates the movement of the main ions toward the cathode during electrodeposition.

**FIGURE 1:**
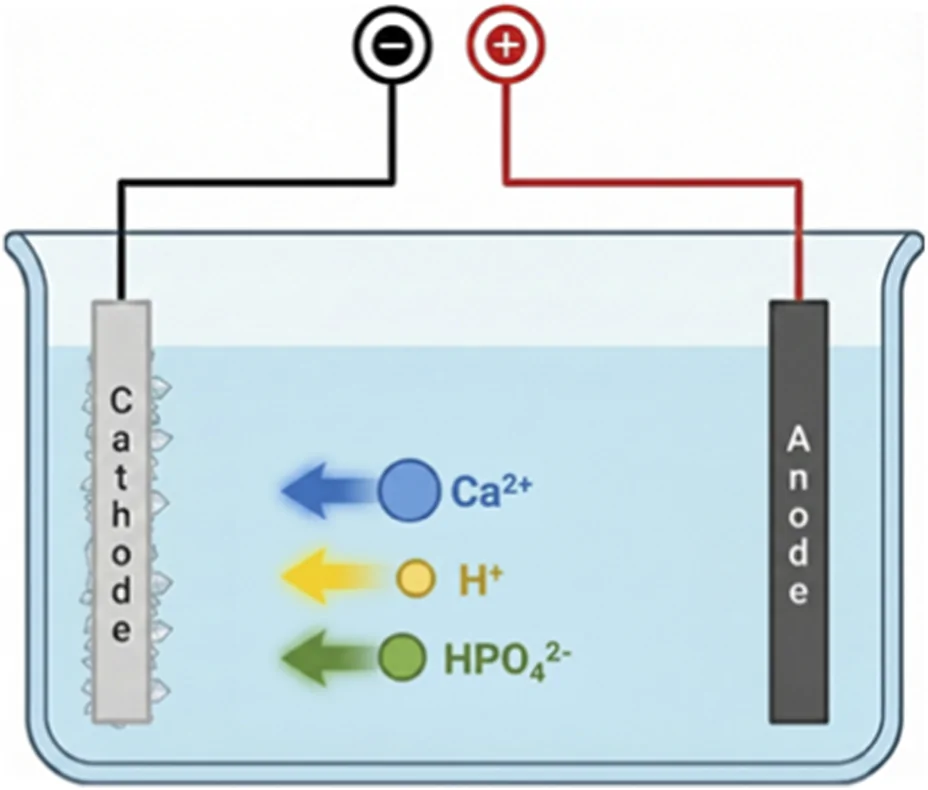
Model of the movement towards the cathode surface of the main ions in the electrolyte.

On the surface of the cathode, calcium ions react with HPO_4_^2-^ and PO_4_^3-^ ions to form dicalcium dihydrogen phosphate (DCPD), also called brushite, according to the reactions described in the Equations 6-9:

$${\text{Ca}}^{2+}+{\text{HPO}}_{4}^{2-}+{2H}_{2}O\to \text{Ca}{\text{HPO}}_{4}\cdot {2H}_{2}O\left(\text{DCPD}\right)$$ (6)

Other form of calcium-phosphates such as octacalcium phosphate (OCP), hydroxyapatite (HA) and tricalcium phosphate (TCP) could also form according to the following reactions:

$$4{\text{Ca}}^{2+}+{\text{HPO}\;}_{4}^{2-}+{2\text{PO}}_{4}^{3-}+2.5H_{2}O\;\to \;{\text{Ca}}_{4}H{\left({\text{PO}}_{4}\right)}_{3}\cdot \;2.5H_{2}O\;\left(\text{OCP}\right)$$ (7)

$$5{\text{Ca}}^{2+}+{3\text{PO}}_{4}^{3}+{\text{OH}}^{-}\to \;{\text{Ca}}_{5}\;{\left({\text{PO}}_{4}\right)}_{3}\;\left(\text{OH}\right)\;\left(\text{HA}\right)$$ (8)

$$3{\text{Ca}}^{2+}+{2\text{PO}\;}_{4}^{3-}\to \;{\text{Ca}}_{3}{\left({\text{PO}}_{4}\right)}_{2}\;\left(\text{TCP}\right)$$ (9)

However, at the beginning of the precipitation process, DCPD formation prevails because of two major factors:

1. Absence of sufficient OH^−^ ions for generation of HA.
2. DCPD is stable at pH < 5 and its precipitation is promoted in the presence of a low amount of OH^−^ ions (acidic condition).

### 2.3 Final preparation of specimens

After electrodeposition, the coated Ti discs were washed with distilled water before entering a clean room for packaging in PA/PE double bags.

Gamma sterilization at 25 kGy was performed on the sealed packages (Group “*Ti-AM + Bru*”).

A second set of uncoated AM discs was also washed, packaged and gamma sterilized under the same conditions. This was used as a reference for *in vitro* testing (Group “*Ti-AM*”).

### 2.4 Chemical analysis by means of ICP

The coating has been analyzed by ICP-OES (inductively coupled plasma-optical emission spectrometry) in order to measure the content of elements and verify the absence of unwanted metals.

For this measurement, 25 cylindrical specimens (31 mm height, 12 mm diameter) were prepared under the same conditions as the specimens used for *in-vitro* testing. Unlike the other analyses, larger samples were used for ICP in order to ensure sufficient coating mass for the detection of trace elements, while ensuring that the deposition process remained identical.

Each specimen was weighted and then immersed into a beaker with 30 ml of distilled water and 1 ml of ultrapure nitric acid (Ultra-pure nitric acid 70%, Sigma Aldrich). Each specimen remained in this solution for 1 min, then put in an oven at 90 °C, for 90 min. After cooling at room temperature, the specimen was re-weighted. Five specimens were immersed in the nitric acid solution in order the increase the amount of diluted coating mass. The obtained solution was finally put into a graduated flask filled up the to 100 ml mark with distilled water.

The procedure was repeated five times in order to have five solutions.

The same procedure was applied also to five blank specimens, i.e. specimens made of the same substrate materials, but not coated with brushite.

Each solution was then measured three times with an ICP-OES Spectro CirOs CCD (SPECTRO Analytical Instruments GmbH, Germany) instrument.

### 2.5 Cell lines

Human MSCs (Lonza Technologies, Walkersville, MD United States) were cultured in mesenchymal stem cell growth medium (MSCGM™ Bullet Kit, Lonza, Walkersville, MD United States) to expand cells without inducing differentiation. The culture medium was changed every 3 days, and cells were split at 80%–90% of confluence using StemPro Accutase (Gibco by Life Technologies, Grand Islands, NY United States). To perform osteogenic differentiation, MSCs were treated with MSC mesenchymal stem cell osteogenic differentiation medium (OM) (hMSC Osteogenic Differentiation Bullet Kit™, Lonza) and to underlined the role of brushite coating on osteogenic differentiation, MSCs were also maintained in basal medium (BM) (hMSCs Basal Growth Medium Bullet Kit^TM^, Lonza).

### 2.6 Establishment of a co-culture system of MSCs and Ti alloys

For transcriptome analysis, MSC cells were seeded (15x10^4^ cells/well in MW 6 well) in OM and BM into 6-well plate (Costa, NY, United States); the day after, hMSC cells were co-cultured for 7 and 14 days, using a transwell system (Corning® Transwell®, 0.4 µm Pore Polycarbonate Membrane Insert), through indirect contact with the coated and uncoated titanium alloy samples (Ti-AM; Ti-AM + Bru), placed at the top of the well in the culture chamber. 500 μl of culture medium was added to each 1 g Ti alloy disc. As an internal experimental control, hMSCs were cultured alone in BM and OM. On 7 and 14 days, the complete medium was removed, hMSCs were washed twice with phosphate buffered saline (PBS) and then immediately processed for RNA extraction.

### 2.7 RNA extraction and real-time PCR

Total RNA was extracted using the commercially available TRIzol Reagent (ThermoFisher Scientific), according to the manufacturer’s instructions. RNA was reverse transcripted to cDNA using the HighCapacity cDNA Reverse Transcription Kit (Applied Biosystems, Fisher Scientific Italia) and relative real-time PCR was performed in duplicates for each data point, using QuantiTect Primers (Qiagen).

Changes in the target mRNA content relative to the housekeeping gene β-actin were determined with the 2^−ΔΔCT^ method (Livak and Schmittgen, 2001).

### 2.8 Transcriptomic analysis

Libraries were generated from 1 µg of RNA using the TruSeq mRNA V2 Prep Kit with Ribo-Zero Gold (Illumina, San Diego, CA, United States). The quality and yield of the prepared libraries were assessed using the Qubit 2.0 fluorometer (Life Technologies, Carlsbad, CA, United States) for quantitative analysis and the TapeStation 4200 system (Agilent Technologies, Santa Clara, CA, United States) for qualitative analysis. Sequencing of the libraries was performed on the NextSeq™ 550 (Illumina, San Diego, CA, United States) with 2 × 76 cycles. Quality control of the raw sequencing data was performed with FastQC (v0.11.9, Babraham Institute). Both low-quality read removal and adapter sequence trimming were performed with trimmomatic (v0.32) (Bolger et al., 2014). The remaining sequences from the cleaning step were mapped to the human reference genome hg19 with STAR (v2.7.0) (Dobin et al., 2013) and transcript amounts were measured with RSEM (v1.3.3) (Li and Dewey, 2011). Gene expression level was normalized by Transcripts Per Million (TPM). To identify differentially expressed genes (DEGs), those transcripts whose fold change was between ≥1.5 and ≤0.6666 with a *p*-value <0.05 were accepted as DEGs. DEGs were analyzed by hierarchical clustering using a “Euclidean distance” distance measure with R functions (v4.1.2).

Standard RNA-seq sequencing quality metrics are provided in Supplementary Figure S3.

The RNA-seq datasets generated during this study have been deposited and are available in the NCBI SRA database with the accession number: PRJNA1506358. The hyperlink to the database is the following: https://www.ncbi.nlm.nih.gov/bioproject/1506358.

### 2.9 Functional enrichment analysis

For functional gene annotation, the obtained sequences were annotated by searching various databases, including Gene Ontology (GO) and the Kyoto Encyclopedia of Genes and Genomes (KEGG). These deregulated genes were subjected to GO and KEGG significant enrichment analyses to identify the biological functions and related metabolic pathways in which these genes are involved. In figures, we only reported the significant top 10 ranked terms.

### 2.10 Analysis of supernatant soluble factors

The level of soluble factors in the cell culture supernatants was analyzed by multiplex-bead immunoassay. Procollagen type I alpha1 (COL1A1), tumor necrosis factor (TNF) alpha, transforming growth factor (TGF)-β1, transforming growth factor (TGF) -β2 and fibronectin (FN) were simultaneously measured by commercially available bead-based sandwich immunoassay kits (Luminex performance Assay multiplex kits, R&D Systems, Minneapolis, MN, United States) according to the manufacturer’s protocols.

Samples or standards were incubated with antibody-coated fluorescent microparticles (50 µl) coated with antibodies specific for each molecule into a 96-well plate After a washing step, samples were incubated with biotinylated antibody cocktail (50 µl) for 1 hour, followed by incubation with fluorescent streptavidin for 30 min. The reaction of sandwich immune complexes on distinct microparticle sets were detected (with) by using the Bio-Plex Protein Array System (Bio-Rad Laboratories, Hercules, CA, United States). The Bio-Plex Manager software version 6.0 (Bio-Rad Laboratories, Hercules, CA, United States) were used for data analyses. Standard curves were employed to quantify soluble factor concentrations by a five-parameter logistic (5 PL) curve-fit. Schematic illustrations, statistical charts and plots were created using BioRender (https://BioRender.com).

### 2.11 Statistical analysis

Statistical analyses were performed using GraphPad Prism version 9.0 (GraphPad Software, San Diego, CA, United States). Data are presented as mean ± standard deviation (SD) from three independent experiments, each performed in triplicate. For qRT-PCR analyses, results are expressed as fold induction (FOI) of Ti-Bru relative to Ti-AM. Differences between experimental groups were evaluated using two-way ANOVA followed by Bonferroni’s multiple comparisons test. For soluble factor quantification, two-way ANOVA followed by Tukey’s multiple comparisons test was used to assess the effects of treatment (Ti-Bru vs. Ti-AM), treatment time, and their interaction. Statistical significance was defined as p < 0.05 (*), p < 0.005 (**), and p < 0.0005 (). Graphical figures were created using BioRender.com.

## 3 Results

### 3.1 Chemical analysis by means of ICP

According to the weight differences recorded before and after immersion in nitric acid, the mass amount of brushite coating dissolved for each specimen ranged between 25.7 mg and 37.3 mg.

Table 1 shows the results of the three ICP measurements for each of the five solutions.

**TABLE 1:** Results of ICP-OES on the five solutions obtained from the brushite-coated specimens.

Element	Unit of measure	Measure	Solution				
			#1	#2	#3	#4	#5
Ca	mg/L	#1	6.76	6.56	8.71	6.79	8.17
		#2	6.80	6.48	8.86	6.84	8.27
		#3	6.92	6.52	9.03	6.77	8.14
P	mg/L	#1	4.58	4.35	5.71	4.81	5.43
		#2	4.56	4.37	5.84	4.80	5.40
		#3	4.50	4.45	5.84	4.79	5.42
Ba	mg/L	#1	0.007	0.005	0.009	0.005	0.006
		#2	0.007	0.005	0.009	0.005	0.006
		#3	0.006	0.005	0.009	0.005	0.006
Cr	mg/L	#1	0.013	0.012	0.018	0.012	0.011
		#2	0.013	0.012	0.019	0.011	0.012
		#3	0.012	0.012	0.018	0.012	0.012
Mn	mg/L	#1	0.120	0.100	0.160	0.100	0.120
		#2	0.012	0.100	0.160	0.100	0.120
		#3	0.012	0.100	0.160	0.100	0.120
Fe	mg/L	#1	0.210	0.200	0.350	0.200	0.180
		#2	0.210	0.200	0.350	0.200	0.180
		#3	0.210	0.200	0.350	0.220	0.180
Ni	mg/L	#1	0.004	0.005	0.016	0.007	0.006
		#2	0.005	0.006	0.014	0.007	0.005
		#3	0.006	0.005	0.016	0.006	0.006
Cu	mg/L	#1	0.140	0.110	0.180	0.120	0.170
		#2	0.140	0.110	0.190	0.110	0.170
		#3	0.140	0.110	0.180	0.110	0.170
Zn	mg/L	#1	0.150	0.130	0.190	0.120	0.190
		#2	0.150	0.130	0.190	0.120	0.190
		#3	0.150	0.130	0.190	0.120	0.190
Cd	mg/L	#1	0.001	0.001	0.001	0.001	0.001
		#2	0.001	0.001	0.001	0.001	0.001
		#3	0.001	0.001	0.001	0.001	0.001

On each solution the concentrations of As, Pb and Hg were also assessed (As: λ = 228.812 nm, Pb: λ = 220.353 nm, Hg: λ = 194.227 nm) but their intensities were below the respective detection limits (As: 0.003 mg/L, Pb: 0.014 mg/L, Hg: 0.02 mg/L).

No other elements were detected in the five solutions obtained from the coated specimens, i.e., their signals were below the detection limits of the instrument used for this quantification.

Finally, ICP measurements of blank samples did not reveal the presence of any of these elements, that is, their signals were below the detection limits of the instrument used for quantification.

### 3.2 Transcriptomic response under osteogenic conditions

#### 3.2.1 Global transcriptomic profiling of MSCs cultured on brushite-coated titanium under osteogenic conditions

Global gene expression profiling by RNA sequencing (RNA-seq) was performed on hMSCs cultured for 7 days on brushite-coated (Ti-AM + Bru) and uncoated titanium (Ti-AM) alloys under osteogenic conditions to characterize early transcriptional responses. Differential expression analysis identified 390 significantly modulated genes (*p* < 0.05, |logFC| ≥ 0.5), including 131 upregulated and 259 downregulated transcripts, while 16,789 genes were not significantly affected. The overall distribution of differentially expressed genes is shown in the volcano plot (Figures 2A). Hierarchical clustering analysis of significantly differentially expressed genes demonstrated a clear separation between Ti-AM + Bru and Ti-AM samples, with consistent clustering within each experimental group (Supplementary Figure 1). The heatmap revealed distinct gene expression patterns, with clusters of genes showing reciprocal regulation between the two conditions, indicating a condition-specific transcriptional response. To further refine this analysis, the top 100 most significantly deregulated genes were selected (Figures 2B). These genes clearly discriminated between Ti-AM + Bru (“case”) and Ti-AM (“control”) samples, confirming the strong impact of brushite surface modification on the transcriptional profile of MSCs. The complete dataset, including baseMean, log2 FoldChange, lfcSE, statistical values, and *p*-values, is provided in Supplementary File 1. As reported in Table 2, upregulated genes contribute to biological processes critical for implant integration, including osteogenesis, inflammation, immune response, energy metabolism, and cell adhesion.

**FIGURE 2:**
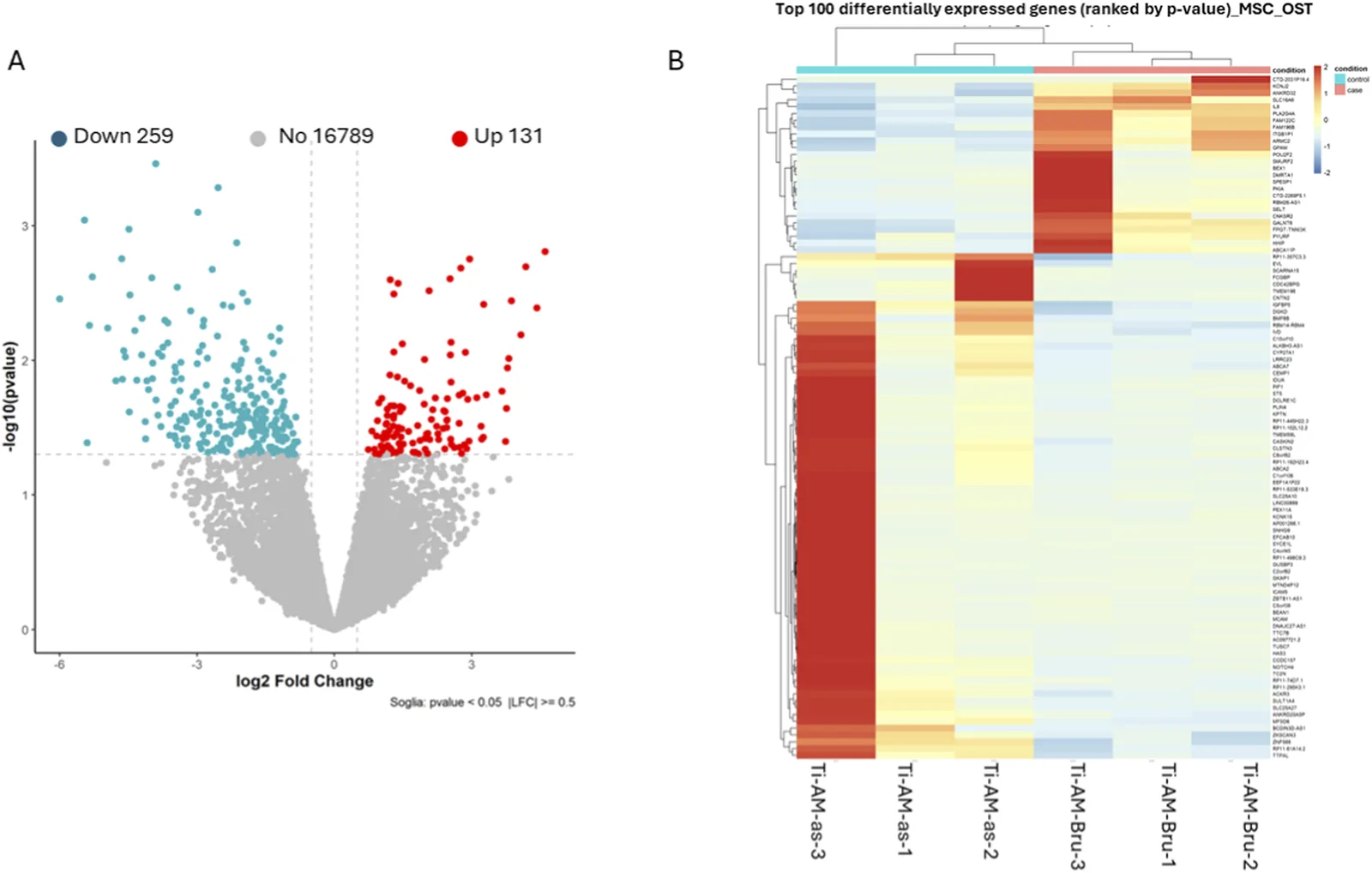
**(A)** Volcano plot of differential gene expression in hMSCs cultured on brushite-coated Ti6Al4V surfaces and uncoated titanium alloy, in osteogenic medium. Volcano plot showing the distribution of differentially expressed genes identified by RNA-seq analysis in hMSCs cultured on brushite-coated Ti6Al4V compared with uncoated titanium controls. The x-axis represents log2 fold change (log2FC), while the y-axis indicates statistical significance expressed as −log10(p-value). Genes significantly upregulated in brushite conditions are shown in red, whereas significantly downregulated genes are shown in cyan; non-significant genes are displayed in gray. Thresholds were set at p < 0.05 and |log2FC| ≥ 0.5. A total of 131 genes were upregulated and 259 downregulated. **(B)** Heatmap of the top 100 most significant differentially expressed genes (ranked by p-value) in hMSCs cultured on brushite-coated and uncoated titanium alloys, under osteogenic conditions for 7 days. Rows represent genes and columns represent individual samples. Gene expression levels are displayed as Z-scores, with red indicating higher expression and blue indicating lower expression. Samples segregate according to experimental condition, indicating distinct gene expression profiles between brushite-coated and control surfaces.

**TABLE 2:** Selected upregulated genes in MSCs cultured on Ti-AM + Bru compared to Ti-AM under osteogenic conditions, with their biological roles and potential contributions to early osseointegration.

Gene	Role	Pro-osteointegrative role in early phase
BMP2	Promotes osteogenic differentiation and bone formation. Crucial for osseointegration	Upregulation of BMP2 indicates activation of osteogenesis, stimulating the differentiation of MSCs into osteoblasts and initiating bone formation at the implant site, which is essential for successful osseointegration
FZD3	Involved in wnt signaling pathway, essential for osteoblast differentiation and bone formation	Upregulation of FZD3 activates the wnt pathway, which is essential for osteoblast differentiation and the promotion of bone regeneration around the Implant, aiding in early osteointegration
SMURF2	Regulates osteoblast differentiation and bone homeostasis, modulating osteogenesis	Upregulation of SMURF2 supports the regulation of osteoblast differentiation, ensuring proper bone formation and remodeling, which is critical for implant stabilization and osseointegration
IL8	Pro-inflammatory cytokine involved in immune cell recruitment and inflammation at the implant site	Upregulation of IL8 contributes to the inflammatory phase by attracting immune cells to the implant site, which helps in tissue repair and infection control. While inflammation is necessary for healing, its resolution is also crucial for osteointegration
OAS1	Part of the immune response, involved in antiviral defense and modulating inflammation	Upregulation of OAS1 activates the innate immune system to protect the implant site, while also controlling inflammation, creating a balanced environment that supports tissue healing and osseointegration
IFIT1	Interferon-induced gene, involved in immune modulation and the antiviral response	Upregulation of IFIT1 enhances the immune response to defend against potential infection at the implant site, while promoting immune regulation to ensure that inflammation does not hinder the osteointegration process
NDUFA9	Part of mitochondrial complex I, involved in cellular energy production necessary for cell proliferation and differentiation	Upregulation of NDUFA9 supports increased energy production, which is vital for cell proliferation and osteoblast differentiation during the early stages of bone healing and implant integration
MTHFD2L	Involved in folate metabolism, crucial for cell division, DNA synthesis, and overall cell function	Upregulation of MTHFD2L facilitates the necessary metabolic processes for cell proliferation and differentiation, ensuring that osteoblasts are able to efficiently form new bone around the implant
SCL20A1	Phosphate transporter essential for bone mineralization, contributing to bone formation around the implant	Upregulation of SLC20A1 enables efficient phosphate transport, which is critical for bone mineralization, promoting the formation of a mineralized bone matrix at the implant site, a key part of osseointegration

Downregulated genes in MSC on Ti-AM + Bru surfaces vs. MSC on Ti-AM surfaces are consistent with early implant healing, reflecting a shift from inflammation toward tissue regeneration and bone formation. This profile indicates activation of osteogenic pathways and resolution of early stress and immune responses, promoting a stable environment for osseointegration. As shown in Table 3, these changes support the expected progression toward successful implant integration.

**TABLE 3:** Selected downregulated genes in MSCs cultured on Ti-AM + Bru compared to Ti-AM under osteogenic conditions, with their biological roles and potential contributions to early osseointegration.

Gene	Role	Pro-osteointegrative role in early phase
BMP8B	Bone morphogenetic protein 8B – involved in osteogenesis and bone formation	Downregulation of BMP8B may indicate that the initial osteogenic signaling is being modulated after early activation, allowing the bone healing process to stabilize and mature
WNT7B	Part of the wnt signaling pathway, critical for osteoblast differentiation and bone formation	Downregulation of WNT7B suggests a shift from early-stage osteogenesis to more regulated and stable bone formation, aiding in the long-term integration of the implant
NOTCH4	A gene involved in cell differentiation, particularly osteoblast differentiation	Downregulation of NOTCH4 implies that the initial osteoblast differentiation phase is transitioning into a more stable bone remodeling phase, essential for implant osseointegration
ICAM5	Intercellular adhesion molecule 5, involved in immune cell adhesion and recruitment	Downregulation of ICAM5 indicates that the acute inflammatory response is subsiding, which is necessary for a balanced immune response, crucial for the successful integration of the implant
SAA2	Serum amyloid A2, an acute-phase protein that is upregulated during inflammation	Downregulation of SAA2 suggests a decrease in inflammation, signaling the transition from the inflammatory phase to tissue repair and remodeling, which is critical for osseointegration
GUSBP3	Involved in immune modulation and cellular stress response	Downregulation of GUSBP3 reflects a reduction in the early stress response, suggesting that the cellular environment is stabilizing and becoming more conducive to bone formation and healing

#### 3.2.2 Pathway enrichment analysis of up- and downregulated genes in osteogenic culture conditions

To provide an integrated view of the transcriptional response, differential gene expression and pathway enrichment analyses were performed on MSCs cultured under the experimental conditions (Figures 3, 4). Upregulated genes were significantly associated with pathways involved in osteogenic commitment, developmental signaling, and metabolic adaptation. Reactome analysis highlighted enrichment of PCP protein localization, Hedgehog pathway activation, AXIN degradation, and regulation of RUNX2, RUNX3, and PTEN, suggesting a coordinated activation of osteogenic and differentiation-related programs. Consistently, KEGG analysis revealed enrichment of complement and coagulation cascades, N-glycan biosynthesis, proteasome activity, and Hedgehog signaling, suggesting the concurrent involvement of immune modulation, protein turnover, and developmental pathways. GO enrichment further supported these findings, showing enrichment of oxidoreductase and phosphatase activities, lipid and protein metabolic processes, mitochondrial electron transport, and oxidative stress–related responses, reflecting early metabolic and functional adaptation to the biomaterial interface.

**FIGURE 3:**
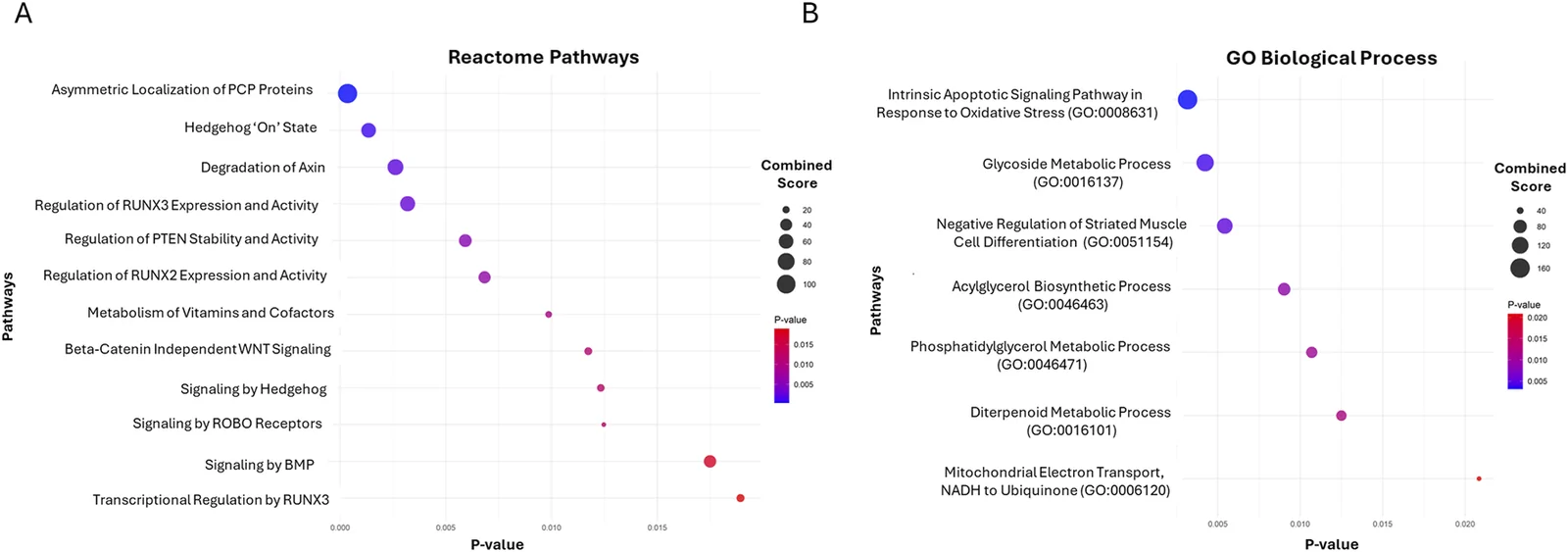
Pathway enrichment analysis of differentially expressed genes (up-regulated) in hMSCs cultured on brushite-coated Ti6Al4V surfaces, in osteogenic medium. **(A)** Reactome pathway enrichment analysis of differentially expressed genes in hMSCs cultured on brushite-coated Ti6Al4V surfaces. Bubble plot showing significantly enriched Reactome pathways identified from differentially expressed genes in hMSCs cultured on brushite-coated Ti6Al4V compared with uncoated titanium controls at 7 days under osteogenic conditions. The y-axis lists enriched pathways, while the x-axis represents enrichment p-values. Bubble size corresponds to the combined enrichment score, and color intensity reflects statistical significance, with lower p-values shown in blue/purple and higher p-values in red. **(B)** GO Biological Process enrichment analysis of differentially expressed genes (up-regulated) in hMSCs cultured on brushite-coated Ti6Al4V surface. Bubble plot showing significantly enriched GO Biological Process pathways identified from differentially expressed genes in hMSCs cultured on brushite-coated Ti6Al4V compared with uncoated titanium controls at 7 days under osteogenic conditions. The y-axis lists enriched pathways, while the x-axis represents enrichment p-values. Bubble size corresponds to the combined enrichment score, and color intensity reflects statistical significance, with lower p-values shown in blue/purple and higher p-values in red.

**FIGURE 4:**
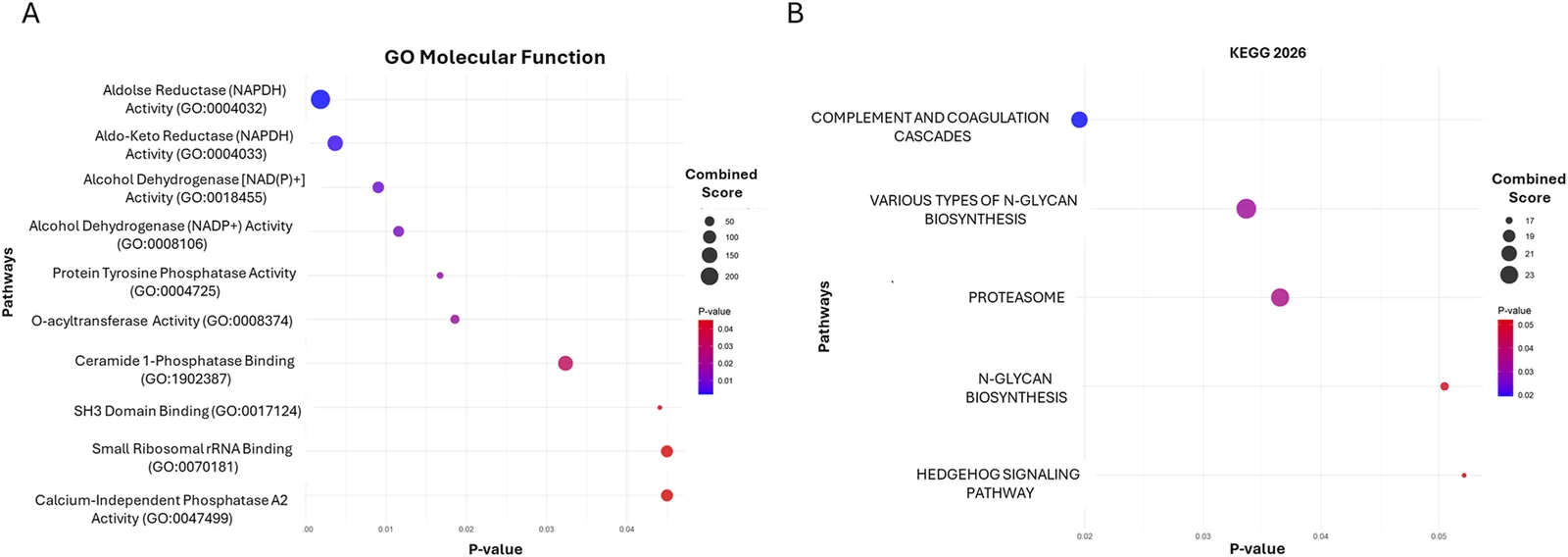
Pathway enrichment analysis of differentially expressed genes (up-regulated) in hMSCs cultured on brushite-coated Ti6Al4V surfaces, in osteogenic medium. **(A)** GO Molecular Function enrichment analysis of differentially expressed genes in hMSCs cultured on brushite-coated Ti6Al4V surface. Bubble plot showing significantly enriched GO Molecular Function pathways identified from differentially expressed genes in hMSCs cultured on brushite-coated Ti6Al4V compared with uncoated titanium controls at 7 days under osteogenic conditions. The y-axis lists enriched pathways, while the x-axis represents enrichment p-values. Bubble size corresponds to the combined enrichment score, and color intensity reflects statistical significance, with lower p-values shown in blue/purple and higher p-values in red. **(B)** Kegg enrichment analysis of differentially expressed genes (up-regulated) in hMSCs cultured on brushite-coated Ti6Al4V surface. Bubble plot showing significantly enriched Kegg pathways identified from differentially expressed genes in hMSCs cultured on brushite-coated Ti6Al4V compared with uncoated titanium controls at 7 days under osteogenic conditions. The y-axis lists enriched pathways, while the x-axis represents enrichment p-values. Bubble size corresponds to the combined enrichment score, and color intensity reflects statistical significance, with lower p-values shown in blue/purple and higher p-values in red.

Conversely, downregulated genes showed enrichment of pathways associated with proliferative signaling, cytoskeletal regulation, immune activation, and cellular stress responses. Reactome analysis identified pathways related to AKT signaling, Rho GTPase cycles (RHOB, RHOF), mismatch repair, and telomere-related pathways, suggesting potential alterations in proliferative activity and cytoskeletal remodeling. KEGG analysis identified enrichment of ABC transporters, while GO terms highlighted decreased DNA exonuclease activity, chemokine receptor signaling, Hsp90 binding, cholesterol efflux, fatty acid transport, and cAMP-responsive processes. Collectively, these results are consistent with a coordinated transcriptional shift characterized by activation of osteogenic and metabolic programs and concomitant attenuation of inflammatory, stress-related, and proliferative pathways, consistent with the establishment of a stable, pro-osteointegrative cellular state at the biomaterial interface (Figures 5, 6).

**FIGURE 5:**
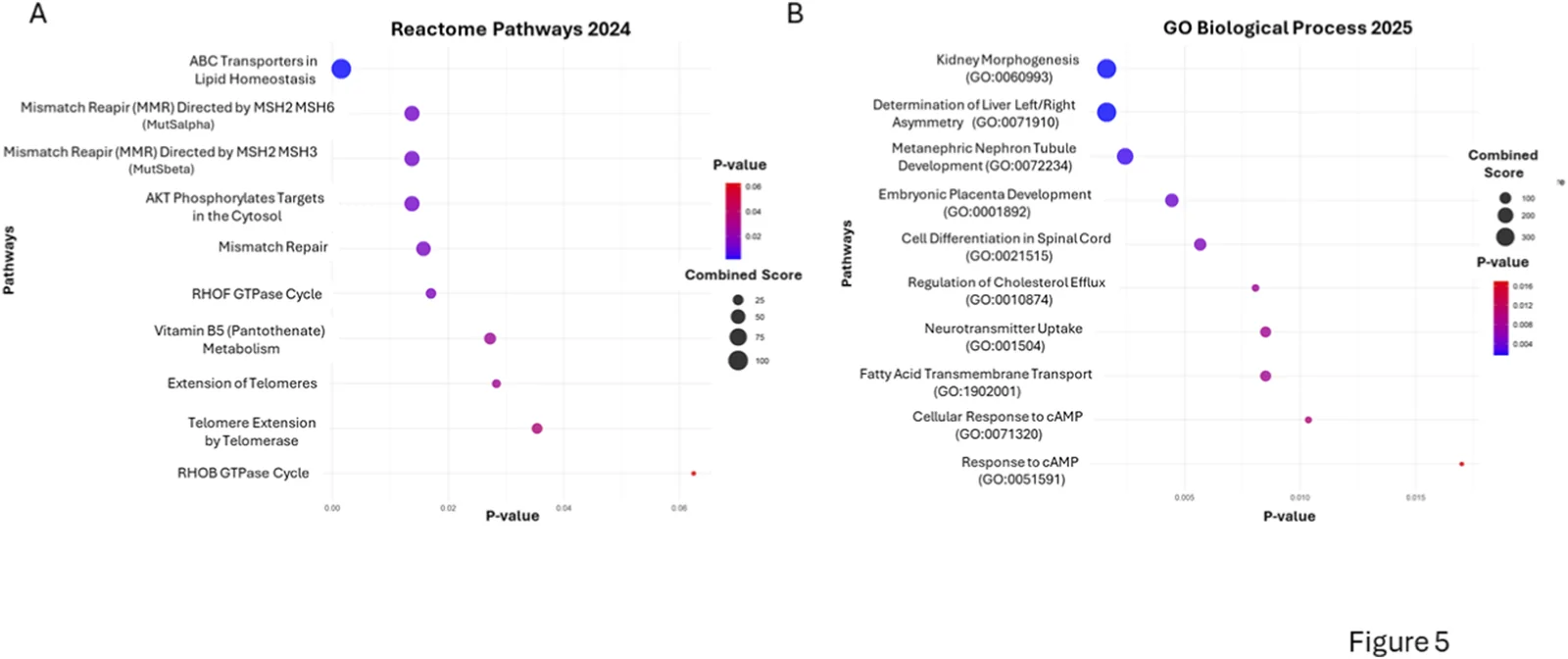
**(A)** Pathway enrichment analysis of differentially expressed genes (down-regulated) in hMSCs cultured on brushite-coated Ti6Al4V surfaces, in osteogenic medium. **(A)** Reactome pathway enrichment analysis of differentially expressed genes in hMSCs cultured on brushite-coated Ti6Al4V surfaces. Bubble plot showing significantly enriched Reactome pathways identified from differentially expressed genes in hMSCs cultured on brushite-coated Ti6Al4V compared with uncoated titanium controls at 7 days under osteogenic conditions. The y-axis lists enriched pathways, while the x-axis represents enrichment p-values. Bubble size corresponds to the combined enrichment score, and color intensity reflects statistical significance, with lower p-values shown in blue/purple and higher p-values in red. **(B)** GO Biological Process enrichment analysis of differentially expressed genes (down-regulated) in hMSCs cultured on brushite-coated Ti6Al4V surface. Bubble plot showing significantly enriched GO Biological Process pathways identified from differentially expressed genes in hMSCs cultured on brushite-coated Ti6Al4V compared with uncoated titanium controls at 7 days under osteogenic conditions. The y-axis lists enriched pathways, while the x-axis represents enrichment p-values. Bubble size corresponds to the combined enrichment score, and color intensity reflects statistical significance, with lower p-values shown in blue/purple and higher p-values in red.

**FIGURE 6:**
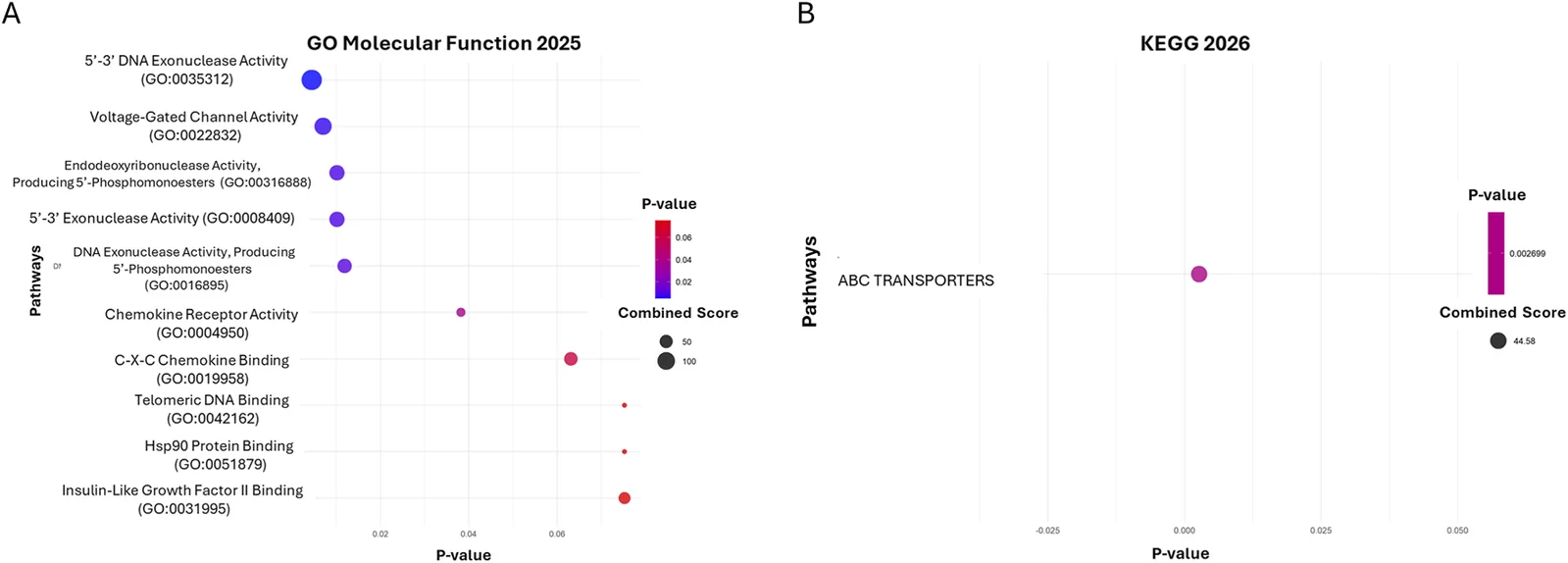
Pathway enrichment analysis of differentially expressed genes (down-regulated) in hMSCs cultured on brushite-coated Ti6Al4V surfaces, in osteogenic medium. **(A)** GO Molecular Function enrichment analysis of differentially expressed genes in hMSCs cultured on brushite-coated Ti6Al4V surface. Bubble plot showing significantly enriched GO Molecular Function pathways identified from differentially expressed genes in hMSCs cultured on brushite-coated Ti6Al4V compared with uncoated titanium controls at 7 days under osteogenic conditions. The y-axis lists enriched pathways, while the x-axis represents enrichment p-values. Bubble size corresponds to the combined enrichment score, and color intensity reflects statistical significance, with lower p-values shown in blue/purple and higher p-values in red. **(B)** Kegg enrichment analysis of differentially expressed genes (down-regulated) in hMSCs cultured on brushite-coated Ti6Al4V surface. Bubble plot showing significantly enriched Kegg pathways identified from differentially expressed genes in hMSCs cultured on brushite-coated Ti6Al4V compared with uncoated titanium controls at 7 days under osteogenic conditions. The y-axis lists enriched pathways, while the x-axis represents enrichment p-values. Bubble size corresponds to the combined enrichment score, and color intensity reflects statistical significance, with lower p-values shown in blue/purple and higher p-values in red.

### 3.3 Transcriptomic response under basal conditions

To evaluate material-driven effects independently of osteogenic induction, global transcriptomic profiling was performed by RNA sequencing (RNA-seq) on MSCs cultured for 7 days on Ti-AM + Bru and Ti-AM under basal conditions. Differential expression analysis identified 613 significantly modulated genes (*p* < 0.05, |log_2_FC| ≥ 0.5), of which 343 were upregulated and 270 downregulated in Ti-AM + Bru compared to Ti-AM, while 15,109 genes were not significantly affected.

The overall distribution of differentially expressed genes is illustrated in the volcano plot (Figure 7A), highlighting a robust transcriptional response driven by the biomaterial surface even in the absence of osteogenic stimulation. Hierarchical clustering of the top 100 most significantly differentially expressed genes revealed a clear separation between Ti-AM + Bru and Ti-AM samples (Figure 7B).

**FIGURE 7:**
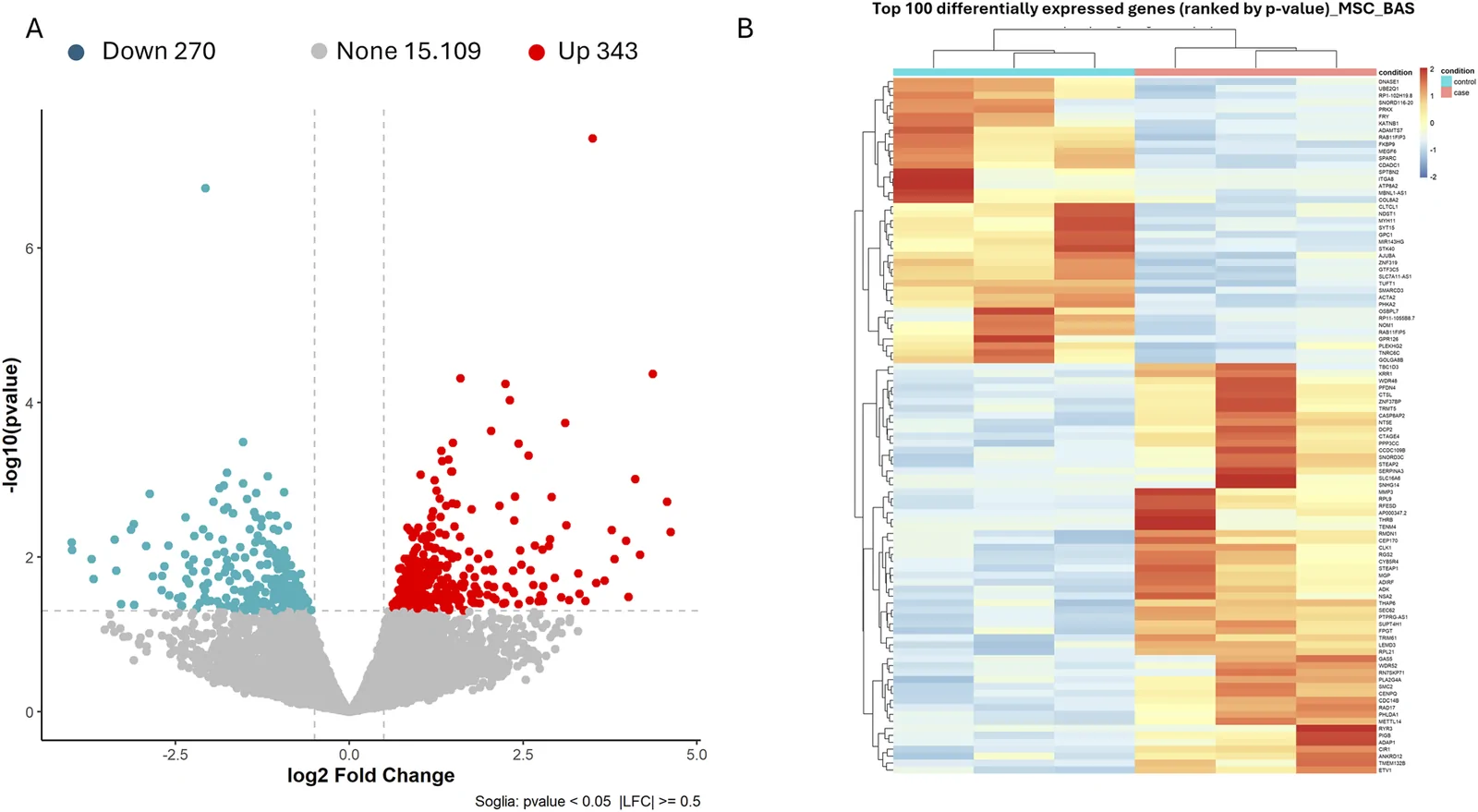
**(A)** Volcano plot of differential gene expression in hMSCs cultured on brushite-coated Ti6Al4V surfaces and uncoated titanium alloy, in basal medium. Volcano plot showing the distribution of differentially expressed genes identified by RNA-seq analysis in hMSCs cultured on brushite-coated Ti6Al4V compared with uncoated titanium controls. The x-axis represents log2 fold change (log2FC), while the y-axis indicates statistical significance expressed as −log10(p-value). Genes significantly upregulated in brushite conditions are shown in red, whereas significantly downregulated genes are shown in cyan; non-significant genes are displayed in gray. Thresholds were set at p < 0.05 and |log2FC| ≥ 0.5. A total of 343 genes were upregulated and 270 downregulated. **(B)** Heatmap of the top 100 most significant differentially expressed genes (ranked by p-value) in hMSCs cultured on brushite-coated and uncoated titanium alloys, under basal conditions for 7 days. Rows represent genes and columns represent individual samples. Gene expression levels are displayed as Z-scores, with red indicating higher expression and blue indicating lower expression. Samples segregate according to experimental condition, indicating distinct gene expression profiles between brushite-coated and control surfaces.

Samples clustered according to surface type, indicating high intra-group consistency. The heatmap showed two major gene clusters with reciprocal expression patterns, with genes upregulated in Ti-AM + Bru consistently downregulated in Ti-AM samples, and vice versa (Supplementary Figure 2). The complete dataset, including baseMean, log2FoldChange, lfcSE, statistical values, and *p*-values, is provided in Supplementary File 2. RNA-seq analysis revealed a broad transcriptional reprogramming in MSCs cultured on brushite-coated titanium under basal conditions, involving genes linked to extracellular matrix remodeling, chemokine signaling, cell cycle regulation, and intracellular trafficking (Table 4). Upregulation of MMP1, MMP3, CXCL1, CXCL5, and CXCL6 was consistent with increased matrix turnover and chemokine activity, reflecting an active cell–material interface. Concurrently, increased expression of DNA damage response and cell cycle regulators (BUB1, CHEK2, RAD17, FANCM) suggests a controlled proliferative state, supported by metabolic and redox gene activation (TXN, ERO1L, HIGD1A), reflecting early bioenergetic adaptation to the biomaterial surface.

**TABLE 4:** Upregulated genes and their potential pro-osseointegration roles under basal conditions.

Gene	Role	Pro-osteointegrative role in early phase
MMP1	Matrix metalloproteinase involved in ECM degradation	Promotes extracellular matrix remodeling, facilitating cell migration and early bone–implant interface adaptation
MMP3	Stromelysin involved in ECM turnover and growth factor activation	Supports matrix remodeling and release of bioactive factors enhancing tissue integration
CXCL1	Chemokine involved in neutrophil recruitment and inflammatory signaling	Modulates early immune signaling and chemotactic environment at the implant interface
CXCL5	Chemokine involved in angiogenesis and leukocyte recruitment	Promotes vascular and immune cell recruitment supporting early healing responses
CXCL6	Chemokine with pro-inflammatory and angiogenic activity	Contributes to immune modulation and early regenerative microenvironment formation
BUB1	Mitotic checkpoint kinase regulating chromosome segregation	Indicates controlled proliferation supporting early tissue expansion and repair
TOPBP1	DNA replication and damage response factor	Ensures genomic stability during active cell cycling in early adaptation phase
CHEK2	DNA damage checkpoint kinase	Regulates stress response and controlled proliferation under biomaterial-induced stress
RAD17	DNA damage checkpoint loading factor	Supports genome integrity during early proliferative response
FANCM	DNA repair and replication stress response protein	Maintains genomic stability during increased cellular activity
TXN	Thioredoxin, redox regulator	Maintains redox balance and supports survival in early oxidative microenvironment
ERO1L	Oxidoreductase involved in disulfide bond formation	Supports protein folding and oxidative metabolism during adaptation
HIGD1A	Mitochondrial stress response protein	Promotes mitochondrial adaptation and cell survival under stress
RAB8B	Vesicle trafficking and membrane transport regulator	Supports intracellular trafficking and membrane remodeling at cell–material interface
RAB27B	Regulates exosome secretion and vesicular transport	Contributes to intercellular communication via extracellular vesicle release
SEC62	Protein translocation into endoplasmic reticulum	Supports protein processing and secretory pathway activation
COG5	Golgi trafficking and glycosylation complex component	Ensures proper protein processing and extracellular matrix organization
COG6	Golgi trafficking and vesicle transport	Supports secretion and ECM-related protein maturation
SUZ12	Epigenetic regulator (PRC2 complex component)	Regulates gene silencing and early lineage commitment programs
HMGA1	Chromatin architectural regulator	Facilitates transcriptional reprogramming and stem cell plasticity
HMGA2	Stemness and proliferation-associated chromatin factor	Promotes early proliferative and undifferentiated cellular state favorable to repair
METTL14	RNA methylation (m6A modification) regulator	Modulates post-transcriptional regulation of genes involved in differentiation and adaptation

In addition, Ti-AM + Bru surfaces induce an early shift toward a quiescent, pro-regenerative MSC state characterized by coordinated modulation of contractile, fibrotic, mechanotransductive, and stress-responsive programs, with reduced ECM stiffening, adhesion strength, and inflammatory signaling (Table 5).

**TABLE 5:** Downregulated genes and their potential pro-osteointegrative roles under basal conditions.

Gene	Role	Functional implication in early response
COL1A2/COL11A1/COL8A2	Fibrillar and basement membrane collagen	Reduced fibrotic ECM deposition and matrix stiffening
SPARC/COMP/SERPINH1/PXDN	ECM organization and collagen maturation	Attenuation of pro-fibrotic matrix assembly
ACTA2/ACTB/ACTG1	Cytoskeletal and contractile proteins	Reduced contractility and stress fiber formation
MYH9/MYH11	Myosin-mediated contractility	Decreased cell tension and myofibroblast-like behavior
FLNA/FLNB	Cytoskeletal scaffolding	Reduced mechanotransduction and structural remodeling
PIEZO1	Mechanosensitive ion channel	Dampened mechanical stress sensing
ITGA11/ITGA8	Cell-ECM adhesion receptors	Reduced strong adhesive/fibrotic adhesion signaling
ADAMTS4/ADAMTS7	ECM proteoglycan remodeling enzymes	Reduced invasive ECM degradation activity
RHOB/ARHGEF network	Rho GTPase signaling	Suppressed cytoskeletal activation and migration
GADD45B/DUSP14/JUND	Stress and MAPK signaling regulators	Reduced inflammatory/stress response activation
TOLLIP	Innate immune signaling regulator	Dampened immune activation
CTNNBIP1/GSK3B	Wnt pathway modulation	Fine-tuning of developmental signaling
NFE2L1	Oxidative stress response TF	Reduced oxidative stress adaptation demand
STK11/SIK1	Metabolic/stress signaling kinases	Shift toward energy homeostasis

### 3.4 Pathway enrichment analysis of up- and downregulated genes in basal culture conditions

To provide an integrated view of the transcriptional response, differential gene expression and pathway enrichment analyses were performed on MSCs cultured under the experimental conditions (Figures 8–11). Integrated pathway analysis indicates an early metabolic and translational activation accompanied by modulation of immune-inflammatory signaling and reduced mechanosensitive adhesion pathways, consistent with a material-driven shift toward a pro-regenerative MSC state (Figures 8–11). In addition, a set of 25 genes was consistently modulated in MSCs cultured on brushite-coated titanium alloy compared to uncoated titanium, both under basal and osteogenic conditions. Most genes showed concordant regulation across conditions, indicating a robust and material-driven transcriptional response. *Downregulated Genes*: Primarily involved in ECM organization, cytoskeletal dynamics, and intracellular signaling. Downregulation occurred in both osteogenic and basal conditions, suggesting reduced activity in these pathways. *Upregulated Genes*: Related to metabolism, immunomodulation, and cell survival, with upregulation observed across both conditions, suggesting enhanced metabolic and survival pathways. A small subset of genes (e.g., ABCA7, SPTBN2) exhibited opposite regulation depending on the culture condition, suggesting a context-dependent response (Table 6).

**FIGURE 8:**
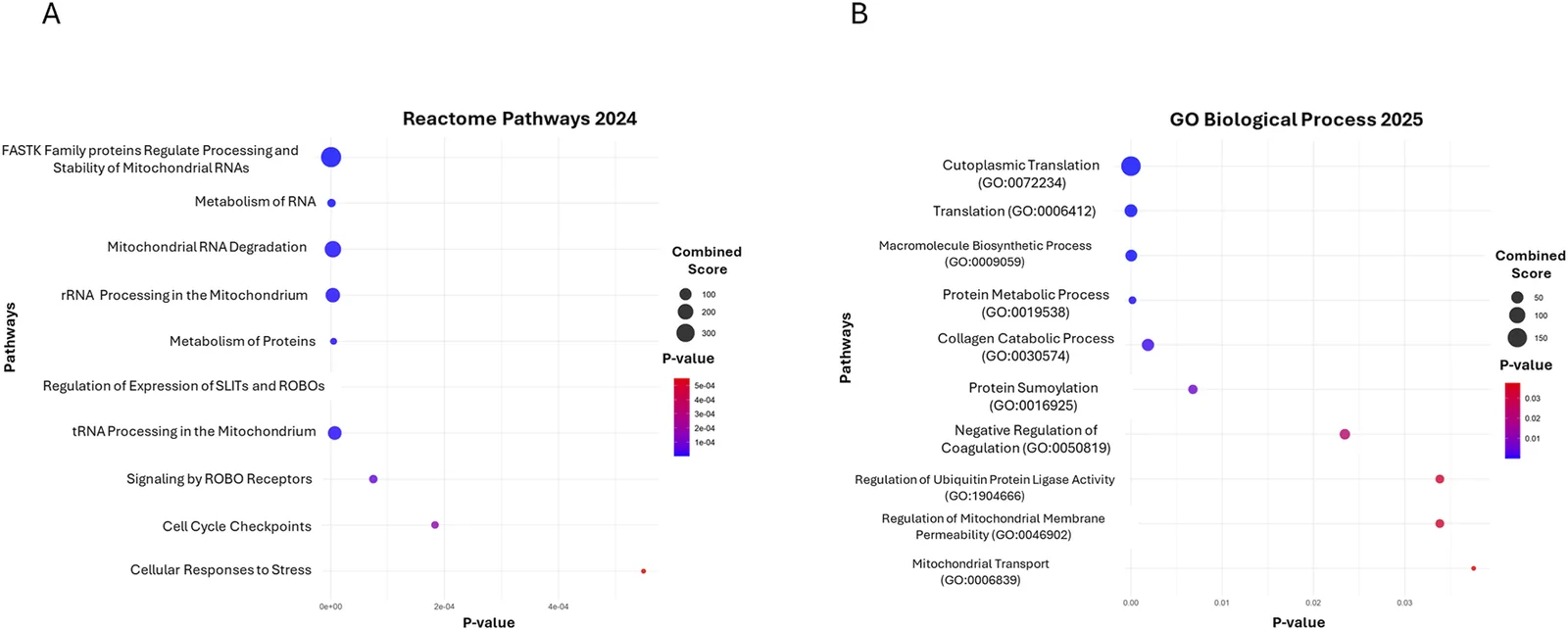
Pathway enrichment analysis of differentially expressed genes (up-regulated) in hMSCs cultured on brushite-coated Ti6Al4V surfaces, in basal medium. **(A)** Reactome pathway enrichment analysis of differentially expressed genes in hMSCs cultured on brushite-coated Ti6Al4V surfaces. Bubble plot showing significantly enriched Reactome pathways identified from differentially expressed genes in hMSCs cultured on brushite-coated Ti6Al4V compared with uncoated titanium controls at 7 days under basal conditions. The y-axis lists enriched pathways, while the x-axis represents enrichment p-values. Bubble size corresponds to the combined enrichment score, and color intensity reflects statistical significance, with lower p-values shown in blue/purple and higher p-values in red. **(B)** GO Biological Process enrichment analysis of differentially expressed genes (up-regulated) in hMSCs cultured on brushite-coated Ti6Al4V surface. Bubble plot showing significantly enriched GO Biological Process pathways identified from differentially expressed genes in hMSCs cultured on brushite-coated Ti6Al4V compared with uncoated titanium controls at 7 days under basal conditions. The y-axis lists enriched pathways, while the x-axis represents enrichment p-values. Bubble size corresponds to the combined enrichment score, and color intensity reflects statistical significance, with lower p-values shown in blue/purple and higher p-values in red.

**FIGURE 9:**
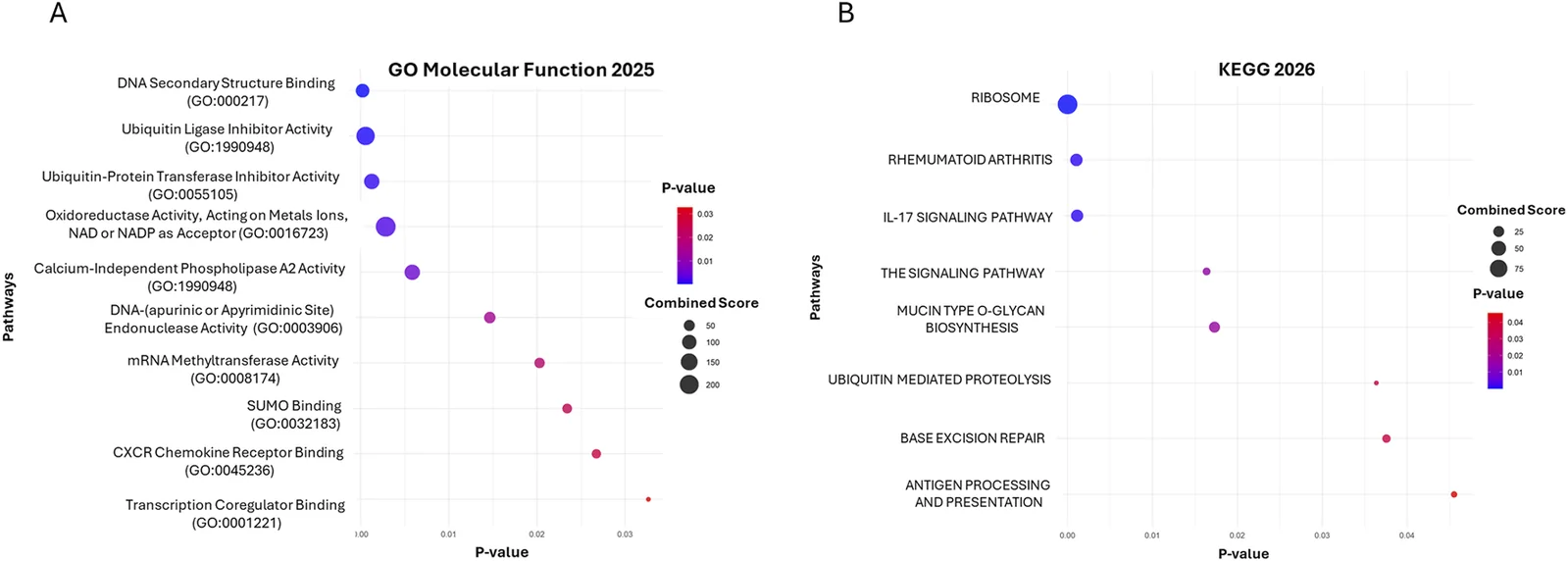
Pathway enrichment analysis of differentially expressed genes (up-regulated) in hMSCs cultured on brushite-coated Ti6Al4V surfaces, in basal medium. **(A)** GO Molecular Function enrichment analysis of differentially expressed genes in hMSCs cultured on brushite-coated Ti6Al4V surface. Bubble plot showing significantly enriched GO Molecular Function pathways identified from differentially expressed genes in hMSCs cultured on brushite-coated Ti6Al4V compared with uncoated titanium controls at 7 days under basal conditions. The y-axis lists enriched pathways, while the x-axis represents enrichment p-values. Bubble size corresponds to the combined enrichment score, and color intensity reflects statistical significance, with lower p-values shown in blue/purple and higher p-values in red. **(B)** Kegg enrichment analysis of differentially expressed genes (up-regulated) in hMSCs cultured on brushite-coated Ti6Al4V surface. Bubble plot showing significantly enriched Kegg pathways identified from differentially expressed genes in hMSCs cultured on brushite-coated Ti6Al4V compared with uncoated titanium controls at 7 days under basal conditions. The y-axis lists enriched pathways, while the x-axis represents enrichment p-values. Bubble size corresponds to the combined enrichment score, and color intensity reflects statistical significance, with lower p-values shown in blue/purple and higher p-values in red.

**FIGURE 10:**
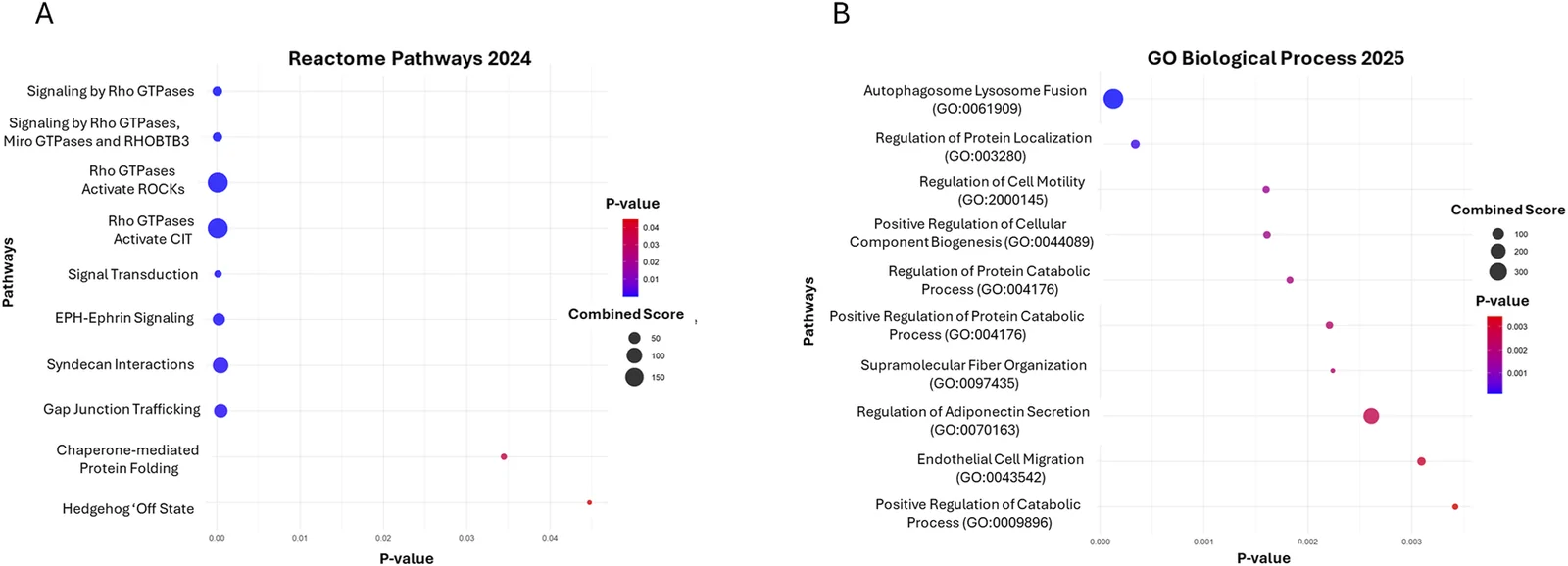
Pathway enrichment analysis of differentially expressed genes (down-regulated) in hMSCs cultured on brushite-coated Ti6Al4V surfaces, in basal medium. **(A)** Reactome pathway enrichment analysis of differentially expressed genes in hMSCs cultured on brushite-coated Ti6Al4V surfaces. Bubble plot showing significantly enriched Reactome pathways identified from differentially expressed genes in hMSCs cultured on brushite-coated Ti6Al4V compared with uncoated titanium controls at 7 days under basal conditions. The y-axis lists enriched pathways, while the x-axis represents enrichment p-values. Bubble size corresponds to the combined enrichment score, and color intensity reflects statistical significance, with lower p-values shown in blue/purple and higher p-values in red. **(B)** GO Biological Process enrichment analysis of differentially expressed genes (down-regulated) in hMSCs cultured on brushite-coated Ti6Al4V surface. Bubble plot showing significantly enriched GO Biological Process pathways identified from differentially expressed genes in hMSCs cultured on brushite-coated Ti6Al4V compared with uncoated titanium controls at 7 days under basal conditions. The y-axis lists enriched pathways, while the x-axis represents enrichment p-values. Bubble size corresponds to the combined enrichment score, and color intensity reflects statistical significance, with lower p-values shown in blue/purple and higher p-values in red.

**FIGURE 11:**
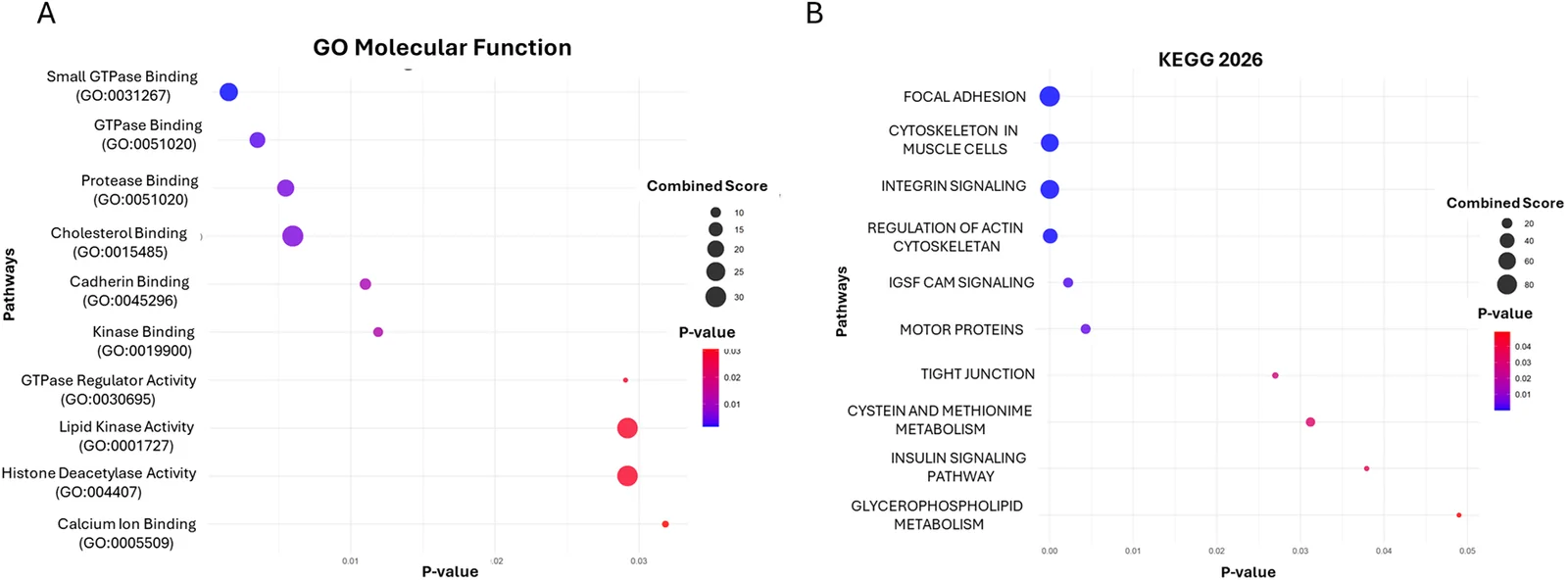
Pathway enrichment analysis of differentially expressed genes (down-regulated) in hMSCs cultured on brushite-coated Ti6Al4V surfaces, in basal medium. **(A)** GO Molecular Function enrichment analysis of differentially expressed genes in hMSCs cultured on brushite-coated Ti6Al4V surface. Bubble plot showing significantly enriched GO Molecular Function pathways identified from differentially expressed genes in hMSCs cultured on brushite-coated Ti6Al4V compared with uncoated titanium controls at 7 days under basal conditions. The y-axis lists enriched pathways, while the x-axis represents enrichment p-values. Bubble size corresponds to the combined enrichment score, and color intensity reflects statistical significance, with lower p-values shown in blue/purple and higher p-values in red. **(B)** Kegg enrichment analysis of differentially expressed genes (down-regulated) in hMSCs cultured on brushite-coated Ti6Al4V surface. Bubble plot showing significantly enriched Kegg pathways identified from differentially expressed genes in hMSCs cultured on brushite-coated Ti6Al4V compared with uncoated titanium controls at 7 days under basal conditions. The y-axis lists enriched pathways, while the x-axis represents enrichment p-values. Bubble size corresponds to the combined enrichment score, and color intensity reflects statistical significance, with lower p-values shown in blue/purple and higher p-values in red.

**TABLE 6:** List of genes concomitantly modulated under basal and osteogenic conditions.

Category	Genes	Regulation pattern	Biological interpretation
Consistently downregulated	ZBTB11-AS1, FNDC1, RP11-73M18.10, KRT80, AHDC1, RP11-295K3.1, KCNH1, DNASE1, DGKD, FRA10AC1, MICAL3, PODN, COL11A1	↓ in OST and BAS	Reduced ECM remodeling, cytoskeletal organization, and signaling activity
Consistently upregulated	NT5E, PDCD10, PHLDA1, CCDC109B, PLA2G4A, LXN, RPGR, PTPN22, SLC16A6, TENM4	↑ in OST and BAS	Enhanced metabolic activity, immunomodulation, and cell survival pathways
Condition-dependent (opposite regulation)	ABCA7, SPTBN2	Opposite in OST vs. BAS	Context-dependent response (lipid transport, cytoskeleton remodeling)

Brushite incorporation was associated with a stable transcriptional signature in MSCs, potentially priming them for osteointegration by modulating key metabolic, immune, and cytoskeletal pathways.

### 3.5 Validation of osteogenic markers at transcriptional and protein levels

The expression of selected osteogenesis-related genes was assessed in MSCs cultured for 7 days under osteogenic conditions on Ti-AM + Bru and Ti-AM substrates. Quantitative gene expression analysis also confirmed the RNA-seq results, showing increased expression of key osteogenic regulators, including RUNX2, BMP2, and SMAD family members, in hMSCs cultured on brushite-coated titanium compared with uncoated titanium (Figure 12).

**FIGURE 12:**
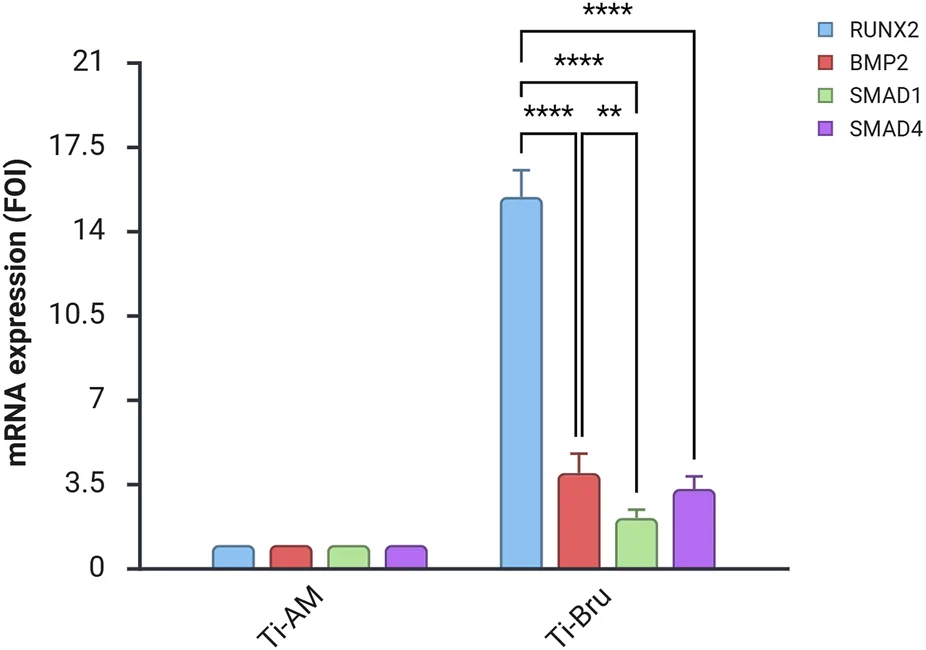
Gene expression analysis. qRT-PCR analysis (n = 3) of the osteogenic genes RNX2, BMP2, SMAD1 and SMAD4. Data were normalized to control (hMSCs co-cultured with Ti-AM alloys in osteogenic conditions) using the 2-ΔΔCt method and expressed as fold of indication (FOI) in gene expression. A two-way ANOVA test was used to evaluate the effect of Ti-AM and Ti-AM+Bru treatments compared to untreated cells (*). All experiments were triplicated, with the data expressed as Mean ± SD; 1 symbol, p < 0.05; 2 symbols, p < 0.005 and 3 symbols, p < 0.0005.

### 3.6 Protein-level validation of the early pro-osteogenic response

Importantly, these transcriptomic findings were further supported by protein-level validation using Bio-Plex multiplex assays. In MSCs cultured on brushite-functionalized titanium alloy under both basal and osteogenic conditions for 7 and 14 days of cultured, a significant upregulation, compared to Ti-AM, of TNFα, procollagen type I alpha 1 (COL1A1), fibronectin (FN), TGF-β1 and TGF-β2 was observed at early time point in both conditions. This effect was particularly pronounced under 7days compared to 14days culture conditions, highlighting the synergistic interaction between surface chemistry and differentiation cues. The early increase in COL1A1 and fibronectin supports rapid extracellular matrix deposition and organization at the biomaterial interface, while the upregulation of TGF-β1 and TGFB-β2 confirms activation of key osteogenic and remodelling pathways (Linsley et al., 2013; Schierano et al., 2021). Interestingly, the increase in COL1A1 protein levels at 7 days occurred despite a transcriptional stabilization or reduction of certain collagen subunits (e.g., COL1A2), suggesting a complex post-transcriptional regulation during early matrix deposition (Pan et al., 2013; Lu et al., 2019). At the same time, TNFα upregulation suggests a tightly regulated early inflammatory response that contributes to cell recruitment and signalling cross-talk required for successful osteointegration (Figure 13). These protein-level data provide critical validation of the transcriptomic results and, importantly, demonstrate that brushite promotes an early upregulation of osteointegrative signalling pathways, rather than merely affecting late-stage differentiation marker (Raimondi et al., 2025; Shaik et al., 2019; Ivanovski et al., 2011). The fact that these effects are already evident at 7 days underscores the role of brushite as an active bifunctional coating capable of accelerating the initial phases of cell commitment and matrix formation.

**FIGURE 13:**
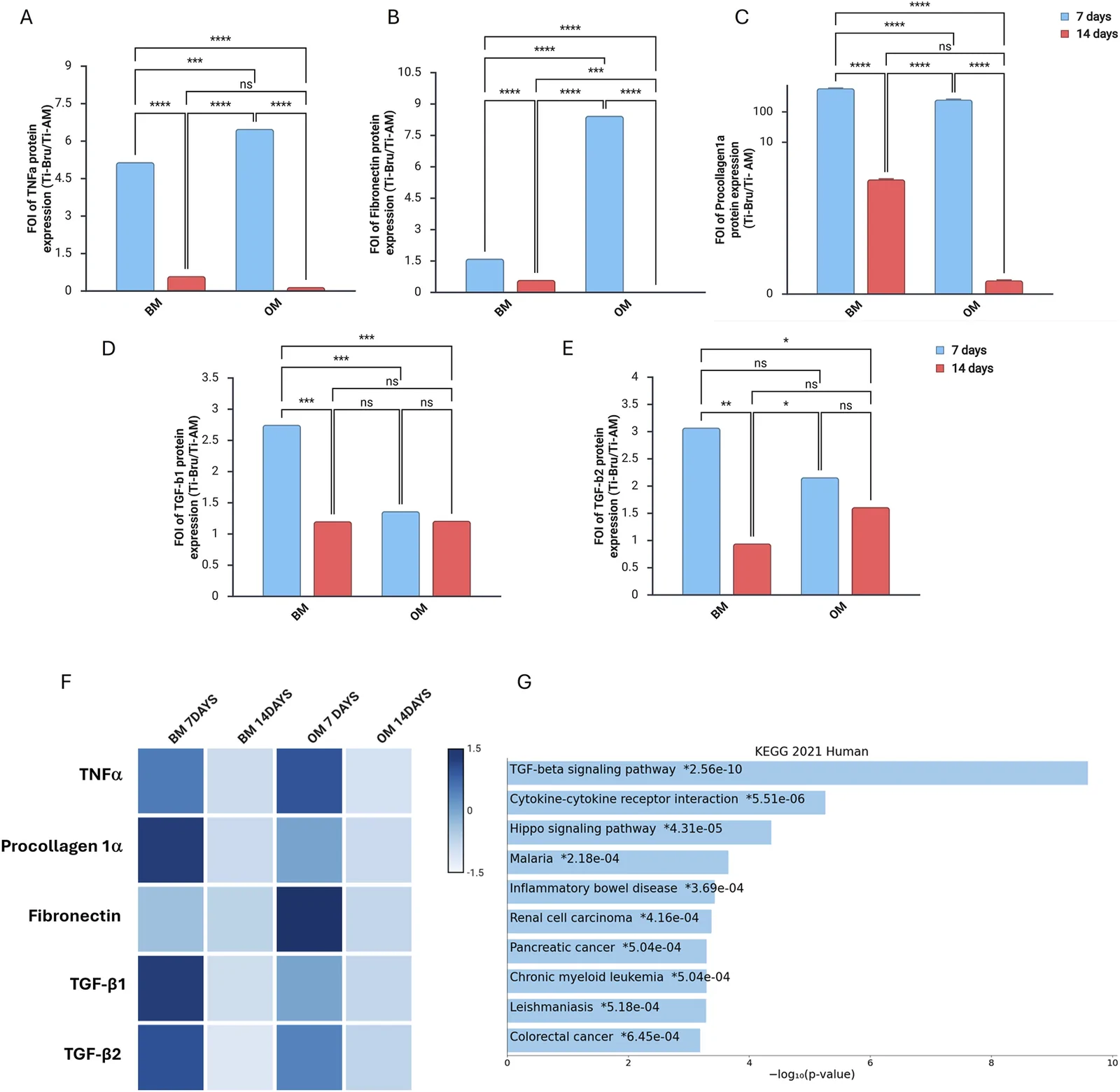
Analysis of protein released from hMSC cells maintained in basal (BM) or osteogenic medium (OM) for 7 anda 14 days on Ti-AM-Bru and Ti-AM Titanium alloy. BioPlex analysis of supernatant soluble factors **(A)** TNFa, **(B)** Fibronectin, **(C)** ProCollagen a1, **(D)** TGFb1, **(E)** TGFb2. Data are expressed as fold of induction (FOI) Ti- Bru vs Ti-AM. A two-way ANOVA and Tukey's multiple comparison test were used to evaluate the effect of Ti-Bru in different colture medium compared to Ti-AM or the effects of treatments during the experimental times (1 symbol, p<0.05; 2 symbols, p < 0.005 and 3 symbols, p < 0.0005). All experiments were triplicated, with the data expressed as Mean ± SD. **(F)** Heat map rapresentation of media of FOI (Ti-Bru vs Ti-AM) of expression of soluble factors released by hMSCs maintained in BM or OM medium for 7 and 14 days. All graphs and statistical analysis are created using https://BioRender.com. **(G)** Enrich analysis of protein released. Bar chart of top enriched terms from KEGG 2021 Human gene set library. The top 10 enriched terms for the input gene set are displayed based on the -log10(p-value), with the actual p-value shown next to each term. The term at the top has the most significant overlap with the input query gene set.

## 4 Discussion

Brushite-coated Ti6Al4V alloys enhance osteogenic activity and biomineralization while modulating the MSC secretome to promote factors critical for bone healing, highlighting their significant potential to improve the biological performance and long-term success of orthopedic prosthetic implants (Raimondi et al., 2025). *In vitro* studies most often assess these effects at later time points (14–21 days) when osteogenic differentiation and matrix mineralization are more pronounced. These late-stage analyses are particularly informative, as they capture the peak of osteogenic activity and provide a comprehensive picture of how brushite coatings influence bone regeneration. However, early-time analyses are equally important, as they reveal initial cellular responses such as adhesion, proliferation, and early signaling events that ultimately set the stage for subsequent differentiation and mineralization (Zhang et al., 2021; Van De Peppel et al., 2017). In addition to the canonical osteogenic culture conditions, studying MSC on brushite-coated titanium under basal conditions can provide valuable insights into the material’s intrinsic ability to modulate cellular behavior. Such analyses allow the assessment of early cellular responses, secretome changes, and potential osteoinductive effects that are independent of exogenous osteogenic stimuli, providing insights into the molecular mechanisms underlying bone regeneration in physiological settings. Building on our previous study demonstrating that brushite-coated Ti6Al4V alloys enhance osteogenic activity and modulate the mesenchymal stem cell secretome, in the present study we focused on the early cellular response to the material. Using NGS analysis, we examined MSCs at 7 days in response to brushite-coated versus uncoated titanium under both osteogenic and basal conditions. We chose the transwell configuration intentionally in order to reproduce the early peri-implant microenvironment, in which recruited MSCs initially respond to diffusible biochemical signals released by the biomaterial before establishing direct contact with the implant surface. This design allowed us to assess the intrinsic effects of the coating (“priming”) on MSC molecular pathways, providing insights into the initial mechanisms that may shape subsequent osteogenic differentiation and bone regeneration. As previously characterized, brushite coatings on Ti6Al4V substrates fabricated via laser powder bed fusion (L-PBF) exhibited the expected brushite phase, characteristic cauliflower-like morphology by SEM, a Ca/P ratio consistent with stoichiometric brushite, rapid dissolution in acidic conditions, adequate adhesion, and an average thickness of ∼27 μm (Raimondi et al., 2025). In particular, the reported dissolution tests conducted according to ASTM F1926 standards demonstrate that the brushite coating almost completely dissolves within 2 hours in HEPES buffer at pH 7.4. Under more acidic conditions (MES buffer, pH 5.5), which can simulate the initial inflammatory microenvironment post-implantation, this rate increases significantly. In the present study, ICP-OES analysis confirmed that Ca and P are the main elements constituting the coating as expected. No elements are present in a concentration higher than the limits indicated by ASTM1609 (F04 Committee, 2026). Trace elements like Fe, Cu, Zn are often found in calcium-phosphates, and the measured concentrations are therefore considered to be acceptable: unsubstituted apatites do not exists in biological systems (Dorozhkin, 2009) and ion substitution impurities are present also in natural bone (Eliaz and Metoki, 2017). Moreover, the bioactivity of calcium phosphate coating can be improved by the presence of such ionic substitutions with different positive effects (Drevet et al., 2023). On each solution toxic elements as, Pb and Hg were not found. For all the cases, concentration for these elements was below the limits imposed by ISO 13779-2 (ISO13779-2:2018: Technical committee: ISO/TC 150/SC 1, 2024) and ASTM F1609 standards (F04 Committee, 2026). All these analytical results provide evidence that the transcriptomic signatures observed in the current study are specifically driven by the bioactive ionic environment generated by the brushite dissolution, primarily the release of calcium and phosphate ions, ensuring that the observed cellular responses were not confounded by impurities. With the material properties established, attention was subsequently directed toward understanding how these features translate into biological functionality. Brushite-coated titanium surfaces elicited a robust and coherent transcriptional response in MSCs under both basal and osteogenic conditions, demonstrating that surface-mediated cues act independently of soluble differentiation factors and are further enhanced in an osteogenic milieu. As first stated, this response was investigated at an early time point, a phase that is often underexplored in favor of later differentiation stages, but which was specifically selected to capture the initial cell–material interaction dynamics preceding stable lineage commitment. This early window of analysis allowed the identification of primary transcriptional adaptations driven directly by surface properties rather than downstream differentiation events, providing mechanistic insight into the earliest phases of MSC reprogramming at the biomaterial interface. The study reveals that brushite-coated titanium surfaces induce a distinct early transcriptional response in MSCs under osteogenic conditions. Transcriptomic profiling by bulk RNA-seq revealed widespread transcriptional remodeling at day 7, as evidenced by the large number of differentially expressed genes (DEGs) identified, with a predominant downregulation, indicating that brushite modification selectively reprograms transcription rather than broadly activating gene expression. Hierarchical clustering and heatmap analyses showed a clear segregation between brushite-coated and uncoated titanium, highlighting a robust material-dependent effect on MSC behavior. Among the upregulated genes, BMP2, FZD3, and SMURF2 are particularly noteworthy, as they are central components of the BMP and Wnt signaling pathways that drive osteoblast differentiation (Zhang et al., 2013; Marcellini et al., 2012). Their early upregulation at 7 days suggests that brushite-coated titanium can prime MSCs, activating key molecular pathways that initiate osteogenic commitment and potentially facilitate subsequent bone integration. Reactome and KEGG pathway enrichment analyses further highlighted the involvement of RUNX2 regulation and Hedgehog signaling, both of which are well-established early drivers of osteoblast differentiation. In agreement with other findings demonstrating the role of SLC20A1-mediated phosphate uptake in ATP-dependent osteogenic signaling (Shih et al., 2014), the observed upregulation of SLC20A1 suggests enhanced commitment toward matrix mineralization, indicating that MSC are primed for matrix deposition and biomineralization. These findings are consistent with previous reports demonstrating that early activation of RUNX2 and Hedgehog pathways, together with enhanced phosphate handling, orchestrates the initiation of osteogenic programs in response to biomaterials (Wang et al., 2025; Komori, 2025). The osteogenic program is characterized by enhanced mitochondrial function and elevated oxygen consumption, reflecting a preferential reliance on oxidative phosphorylation over glycolysis. This metabolic transition provides the energetic support necessary for matrix deposition and mineralization (Smith and Eliseev, 2021). The transcriptional profile revealed increased expression of metabolic genes such as NDUFA9 and MTHFD2L, suggesting a shift toward elevated mitochondrial activity, which is essential to support osteogenic differentiation and matrix production. Concurrently, the upregulation of immune-related genes including IL8, OAS1, and IFIT1 is compatible with a controlled inflammatory response, consistent with reports that early, modulated inflammation is necessary to orchestrate bone healing and facilitate implant integration (Jalloob and HandoolAlfattli, 2025). Together, these findings align with the concept that brushite-coated titanium not only primes MSCs for osteogenesis but also coordinates metabolic and immune pathways critical for successful early-stage bone regeneration. Validation by qRT-PCR at 7 days under osteogenic conditions confirmed the upregulation of key osteogenic regulators, including RUNX2, BMP2, SMAD1, and SMAD4, corroborating the transcriptomic evidence of an early osteogenic priming effect exerted by brushite-coated titanium surfaces. Although NOTCH4, WNT7B, and BMP8B were downregulated, this finding should not be interpreted as an overall inhibition of the corresponding signaling pathways. Instead, changes in the expression of individual ligands may represent a temporal regulatory mechanism accompanying pathway activation. Notably, despite the early downregulation of BMP8B, the higher expression of BMP2 and SMAD signaling components, together with the enrichment of the BMP pathway, implies a significant osteogenic response during the early phases of osseointegration.

In particular, the reduction of NOTCH4 aligns with reports that reduced Notch signaling facilitates the exit from a progenitor-like state and supports osteoblast maturation (Engin et al., 2008); notably, *in vitro* studies have demonstrated that Notch signaling can exert both stimulatory and inhibitory effects on stromal cell osteoblastic differentiation, depending on factors such as cell type, differentiation stage, and the timing of pathway activation (Luo et al., 2019). The observed suppression of AKT signaling, Rho GTPase activity, and DNA mismatch repair pathways indicates reduced proliferative and stress responses (Tanaka et al., 2018; Meyers et al., 2005; Zhu et al., 2024; Huang et al., 2007; Lai et al., 2024), while concomitant downregulation of immune-related genes (SAA2, ICAM5) suggests attenuation of early inflammatory signaling, collectively consistent with stabilization of a differentiated state (Thomas and Jaganathan, 2022; Xu et al., 2023). Taken together, these findings support a model where brushite-coated titanium surfaces promote, in osteogenic condition, an early transition from a reactive, proliferation-associated phenotype to a more metabolically active and differentiation-oriented state. This transition, marked by the activation of osteogenic signaling and immune modulation, is crucial for early osseointegration. Importantly, these effects occur at an early time point (7 days), emphasizing the role of surface chemistry in directing MSC fate before the full activation of mature osteogenic markers.

Under basal conditions, brushite-coated titanium surfaces also induced a distinct transcriptional response in MSCs, independent of osteogenic stimulation. RNA-seq analysis revealed a broad transcriptional reprogramming, marked by the upregulation of genes associated with extracellular matrix (ECM) remodeling, chemokine signaling, and intracellular trafficking. The upregulation of MMP1, MMP3, CXCL1, CXCL5, and CXCL6 suggests potential increased matrix turnover and chemokine-mediated signaling, reflecting an active cell-material interface. Indeed, biomaterials that induce upregulation of specific MMPs and CXCLs can effectively promote MSC-mediated bone regeneration by optimizing the local environment for both mineralization and vascularization (Shaik et al., 2019; Li et al., 2025; Khoswanto, 2023). Enrichment of genes involved in cell cycle regulation and DNA damage response (BUB1, CHEK2, RAD17) suggests a highly proliferative yet tightly regulated cellular state (Shaikh et al., 2023). These genes play essential roles in preserving genomic integrity during the rapid expansion and subsequent differentiation of MSCs into osteoblasts. The increased expression of metabolic and redox-related genes (e.g., TXN, ERO1L, HIGD1A) suggests an early bioenergetic adaptation, supported by enrichment of mitochondrial pathways. Pathway enrichment also highlighted a dominant signature of translational activation, with strong enrichment of ribosomal activity and macromolecule biosynthesis, indicating increased biosynthetic capacity (Lee et al., 2014; Li et al., 2017). In parallel, multiple datasets revealed activation of stress-response and regulatory pathways, including ubiquitin-mediated proteolysis, SUMOylation, and DNA repair mechanisms, suggesting tight control of protein turnover and genomic stability. Furthermore, the activation of inflammatory pathways, such as IL-17 and TNF signaling, points to early and critical mediator in immunomodulatory processes (Sjakste and Riekstiņa, 2021; Pan et al., 2022; Liu et al., 2022; Park et al., 2024; Hess et al., 2009). Downregulated genes may be associated with attenuation of focal adhesion, integrin signaling, and actin cytoskeleton organization. The attenuation of Rho GTPase signaling suggests reduced cytoskeletal dynamics and mechanotransduction, further supported by decreased enrichment of EPH-ephrin signaling and intercellular communication pathways. Taken together, these findings support the hypothesis that brushite coating induces a transcriptional shift in basal conditions characterized by enhanced metabolic, translational, and biosynthetic activity, coupled with suppressed adhesion and cytoskeletal signaling networks. Moreover, even at baseline, the material seems to elicit a biologically active interface rather than a passive cellular state. Our results reveal a sophisticated, context-dependent regulation of MSC cell-cycle dynamics. Under basal conditions, the brushite surface seems to promote a controlled proliferative state (marked by BUB1 and CHEK2 upregulation), likely facilitating initial colonization of the 3D-printed scaffold. Conversely, upon exposure to osteogenic cues, we observed a coordinated reduction of proliferative pathways (including AKT signaling and Rho GTPases), indicating a interesting shift toward terminal differentiation. This material-driven priming effect appears to predispose the cells for rapid extracellular matrix deposition, as further evidenced by increased COL1A1 and TGF-β1/2 secretion at the protein level, even prior to the onset of late-stage mineralization markers (Pan et al., 2013; Lu et al., 2019). Comparison between osteogenic and basal conditions revealed a consistent set of 25 genes modulated across both environments, representing a material-dependent transcriptional signature independent of differentiation cues. This set includes downregulated genes related to ECM organization and cytoskeletal dynamics, and upregulated genes involved in metabolism, immunomodulation, and cell survival (e.g., NT5E, PDCD10, PLA2G4A, SLC16A6). A small subset of genes displayed condition-dependent regulation, reflecting a context-specific response to the surface modification. Overall, these results suggest that brushite-coated titanium surfaces may induce an early adaptive state in MSCs, characterized by increased metabolic and biosynthetic activity, controlled inflammatory signaling, and reduced mechanotransduction. This transcriptional signature appears to prime the cells for subsequent osteogenic commitment under inductive conditions, positioning brushite as a promising material for enhancing the early stages of osseointegration and improving titanium-based implant performance. The transcriptional profile observed at 7 days suggests that brushite-coated titanium-alloy surfaces do not yet induce complete osteogenic differentiation, but rather promote an early cellular state that predisposes MSCs toward subsequent osteogenic commitment. The combination of enhanced metabolic and biosynthetic activity, controlled inflammatory signaling, and reduced mechanotransduction may define an early priming state preceding overt osteogenic differentiation. Since most studies primarily investigate late-stage osteogenesis and mineralization, this early adaptive transcriptional program has likely remained underexplored. These transcriptomic findings were further supported by Bio-Plex multiplex analysis, confirming that the early transcriptional program induced by brushite-coated titanium surfaces is translated into a functional protein output (Hamed et al., 2023; Wall et al., 2009). MSCs cultured on brushite-functionalized titanium showed increased secretion of extracellular matrix components (COL1A1 and FN1), osteogenic and tissue-remodeling factors (TGF-β1 and TGF-β2), and a moderate increase in TNF-α under both basal and osteogenic conditions (Linsley et al., 2013).

The enhanced production of matrix-related proteins supports the establishment of a structurally competent extracellular environment favorable early osteointegration, while the upregulation of TGF-β signaling further suggests activation of pathways involved in MSC commitment and matrix maturation (Barradas et al., 2013). In parallel, the moderate increase in TNF-α is consistent with a controlled pro-inflammatory response known to contribute to the early stages of bone healing and implant integration (Lee et al., 2021; Ince et al., 2008; Huo and Yue, 2020). Importantly, these effects were observed even in the absence of exogenous osteogenic stimuli, indicating that brushite exerts an intrinsic bioactive effect on MSC behavior. Overall, the Bio-Plex data reinforce the transcriptomic evidence, supporting the hypothesis that brushite-coated titanium surfaces promote a primary adaptive and pro-osteogenic cellular state that may facilitate subsequent osteointegration. Overall, the present study highlights the importance of early transcriptomic adaptations in defining biomaterial-driven osteogenic commitment and identifies brushite coatings as promising modulators of the initial phases of bone–implant integration.

## 5 Conclusion

In conclusion, this study identifies brushite-coated Ti6Al4V as a highly active bio-interface capable of modulating early MSC transcriptional programs well before overt differentiation occurs. Our findings highlight a critical 7-day regulatory window where the coating triggers a robust ‘priming’ state characterized by enrichment of genes related to pro-osteogenic signaling (BMP, Wnt, Hedgehog), metabolic/mitochondrial reprogramming, and a controlled immunomodulatory response Notably, the identification of a stable 25-gene core signature across both basal and osteogenic conditions demonstrates that brushite possesses intrinsic bioactivity independent of soluble biochemical cues. These transcriptomic insights, validated by functional protein secretion data, confirm that brushite does not act as a passive scaffold but as a dynamic driver of cell behavior. These findings emphasize the importance of early molecular events in biomaterial-driven osseointegration and provide strong support for the clinical application of brushite-functionalized surfaces in high-performance orthopedic and dental implants.

## Data Availability

Transcriptomic data have been deposited and are available in the NCBI SRA database with the accession number: PRJNA1506358: https://www.ncbi.nlm.nih.gov/bioproject/1506358.

## Supplementary material

SUPPLEMENTARY FIGURE 1 Heatmap of significantly differentially expressed genes in hMSCs cultured on brushite-coated Ti6Al4V surfaces, in osteogenic culture conditions. Hierarchical clustering heatmap showing the expression profiles of 374 significantly differentially expressed genes identified by RNA-seq analysis in hMSCs cultured on brushite-coated Ti6Al4V (case) compared with uncoated titanium controls (control) under osteogenic conditions at 7 days. Rows represent genes and columns represent biological replicates. Gene expression values are displayed as normalized Z-scores, with red indicating higher expression and blue indicating lower expression relative to the mean expression level across samples. Unsupervised hierarchical clustering revealed a clear segregation between brushite-coated and control samples, indicating a distinct transcriptional response induced by the biomaterial surface.

SUPPLEMENTARY FIGURE 2 Heatmap of significantly differentially expressed genes in hMSCs cultured on brushite-coated Ti6Al4V surfaces, in basal culture conditions. Hierarchical clustering heatmap showing the expression profiles of 604 significantly differentially expressed genes identified by RNA-seq analysis in hMSCs cultured on brushite-coated Ti6Al4V (case) compared with uncoated titanium controls (control) under basal conditions at 7 days. Rows represent genes and columns represent biological replicates. Gene expression values are displayed as normalized Z-scores, with red indicating higher expression and blue indicating lower expression relative to the mean expression level across samples. Unsupervised hierarchical clustering revealed a clear segregation between brushite-coated and control samples, indicating a distinct transcriptional response induced by the biomaterial surface.
